# Supplementary material for: Genome-wide functional analysis of phosphatases in the pathogenic fungus Cryptococcus neoformans
Source: Nat Commun. 2020 Aug 24;11:4212. doi: 10.1038/s41467-020-18028-0 (PMC7445287; doi:10.1038/s41467-020-18028-0)
Supplement: Supplementary file 1 — Supplementary Information [file 41467_2020_18028_MOESM1_ESM.pdf]

Supplementary Information

Genome-wide functional analysis of phosphatases in the pathogenic fungus *Cryptococcus neoformans*

Jin *et al*.

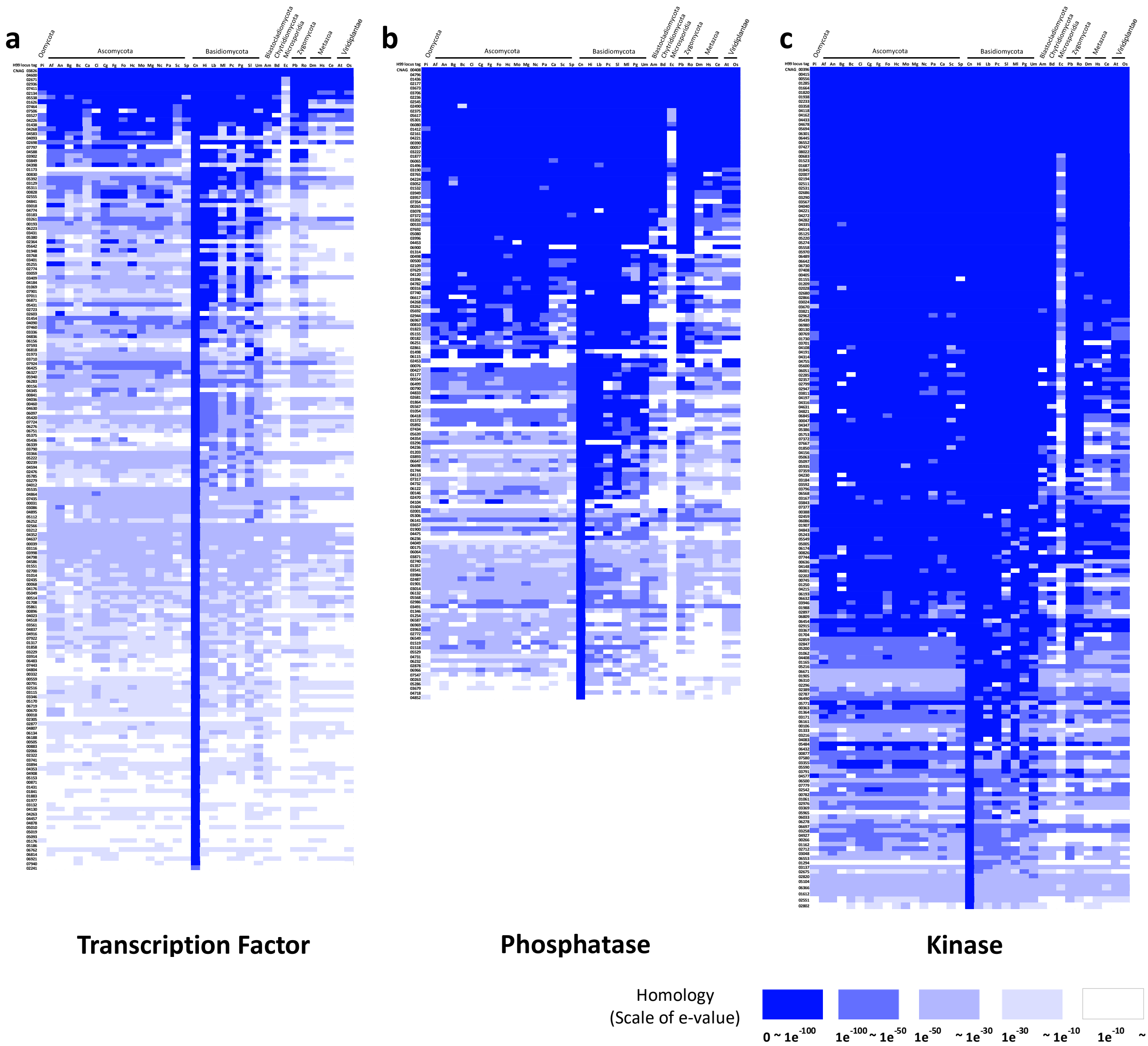

Supplementary Figure 1. BLAST matrix analysis of transcription factors, kinases, and phosphatases in *C. neoformans*. Comparative BLAST matrix analysis performed using the Comparative Fungal Genomics Platform (http://cfgp.riceblast.snu.ac.kr). (**a**) BLAST matrix of 178 *C. neoformans* transcription factors identified by a previous study[1]. (**b**) BLAST matrix of 139 *C. neoformans* phosphatases identified by the current study. (**c**) BLAST matrix of 183 *C. neoformans* kinases identified by a previous study[2].

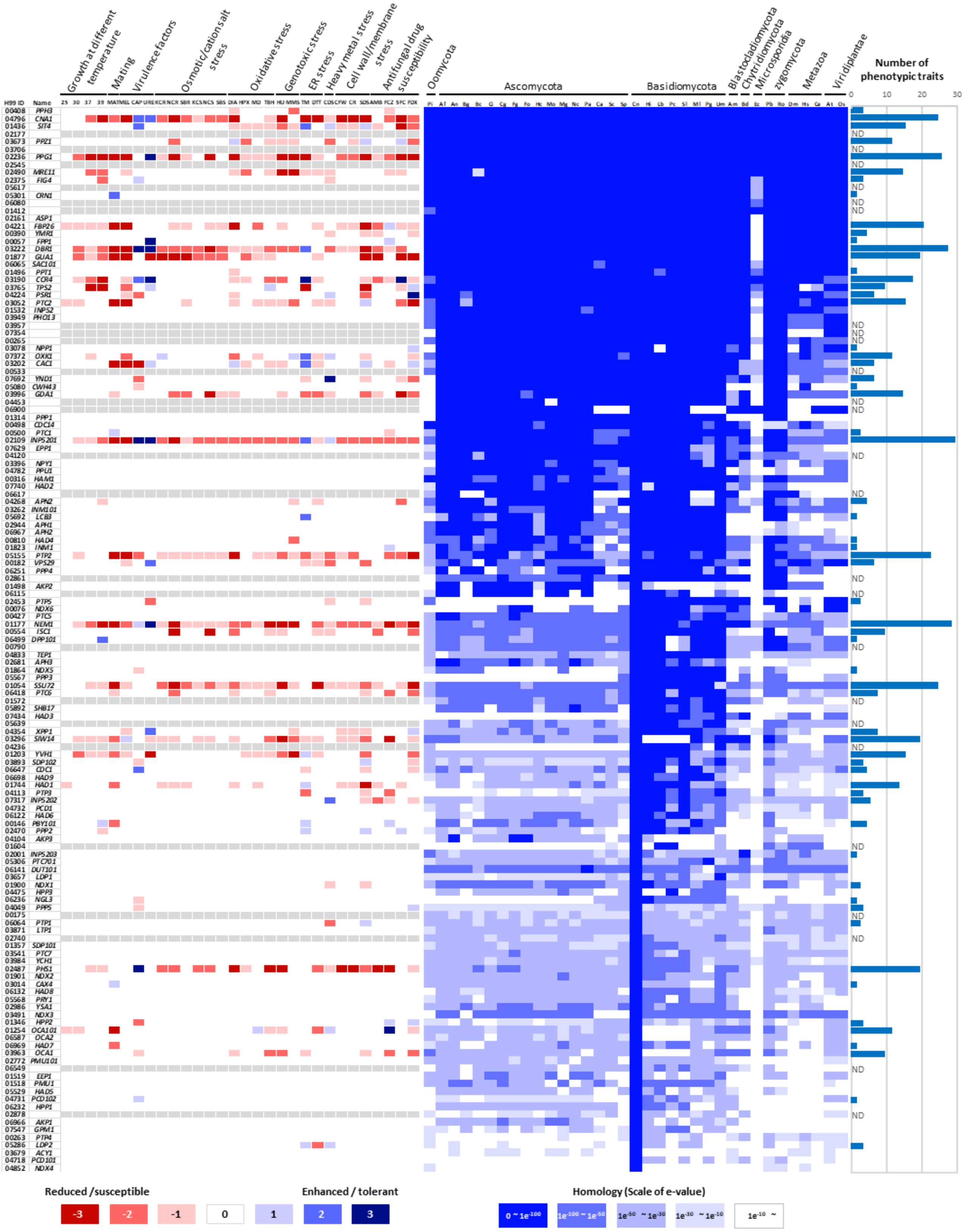

Growth at different temperature
Mating
Virulence factors
Osmotic/cation salt stress
Oxidative stress
Genotoxic stress
ER stress
Heavy metal stress
Cell wall/membrane stress
Antifungal drug susceptibility
Oomycota
Ascomycota
Basidiomycota
Blastocladiomycota
Chytridiomycota
Microsporidia
Zygomycota
Metazoa
Viridiplantae
Number of phenotypic traits
0
10
20
30
H99 ID
Name
ND
Reduced /susceptible
-3
-2
-1
0
1
2
3
Enhanced / tolerant
Homology (Scale of e-value)

Supplementary Figure 2. Phylogenetic relationship and phenotypic traits of phosphatases in *C. neoformans*. BLAST matrix comparative search for each phosphatase was performed using the Comparative Fungal Genomics Platform (http://cfgp.riceblast.snu.ac.kr). In vitro phenotypic traits were examined under 30 different growth conditions and scored on a 7-point scale (-3: strongly reduced/susceptible, -2: moderately reduced/susceptible, -1: weakly reduced/susceptible, 0: wild-type like, +1 weakly enhanced/tolerant, +2: moderately enhanced/tolerant, +3: strongly enhanced/tolerant). All phenotypic data are available in the *Cryptococcus neoformans* Phosphatase Phenome Database (http://phosphatase.cryptococcus.org/). More than three biologically independent experiments were performed for each phenotypic trait. Abbreviations: 25, 25°C; 30, 30°C; 37, 37°C; 39, 39°C; CAP, capsule production; MEL, melanin production; URE, urease production; MAT, mating; HPX, hydrogen peroxide; TBH, *tert*-butyl hydroperoxide; MD, menadione; DIA, diamide; MMS, methyl methanesulphonate; HU, hydroxyurea; 5FC, 5-flucytosine; AMB, amphotericin B; FCZ, fluconazole; FDX, fludioxonil; TM, tunicamycin; DTT, dithiothreitol; CDS, cadmium sulphate; SDS, sodium dodecyl sulphate; CR, Congo red; CFW, calcofluor white; KCR, YPD+1.5 M KCl; NCR, YPD+1.5 M NaCl; SBR, YPD+2 M sorbitol; KCS, YP+1 M KCl; NCS, YP+1 M NaCl; SBS, YP+2 M sorbitol.

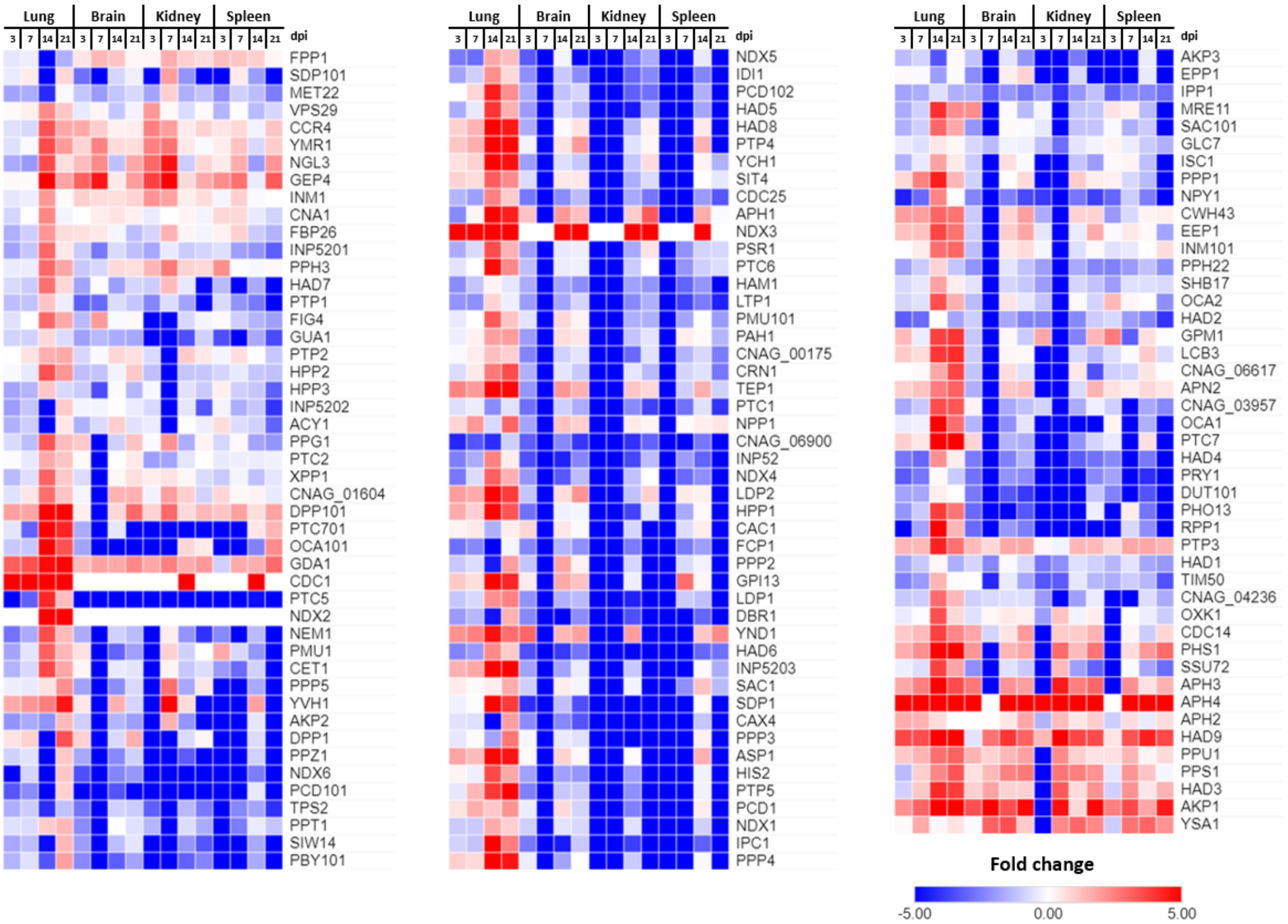

Supplementary Figure 3. *In vivo* gene expression profiling of phosphatases in *C. neoformans*. The *in vivo* gene expression level of 139 phosphatases was monitored using nCounter (NanoString) with RNA samples obtained from wild-type *C. neoformans* strain (H99S)-infected mice organs (lungs, brain, spleen, and kidneys) and gene-specific probes listed in Supplementary Data 7. Three mice were sacrificed 3, 7, 14, or 21 days post-infection (dpi). Infected organs were recovered and lyophilised. Dried organs were homogenised, and total RNA was extracted by using a commercial RNA extraction kit. Hierarchical phenotypic clustering of phosphatases was performed with one minus Pearson correlation using Morpheus (https://software.broadinstitute.org/morpheus).

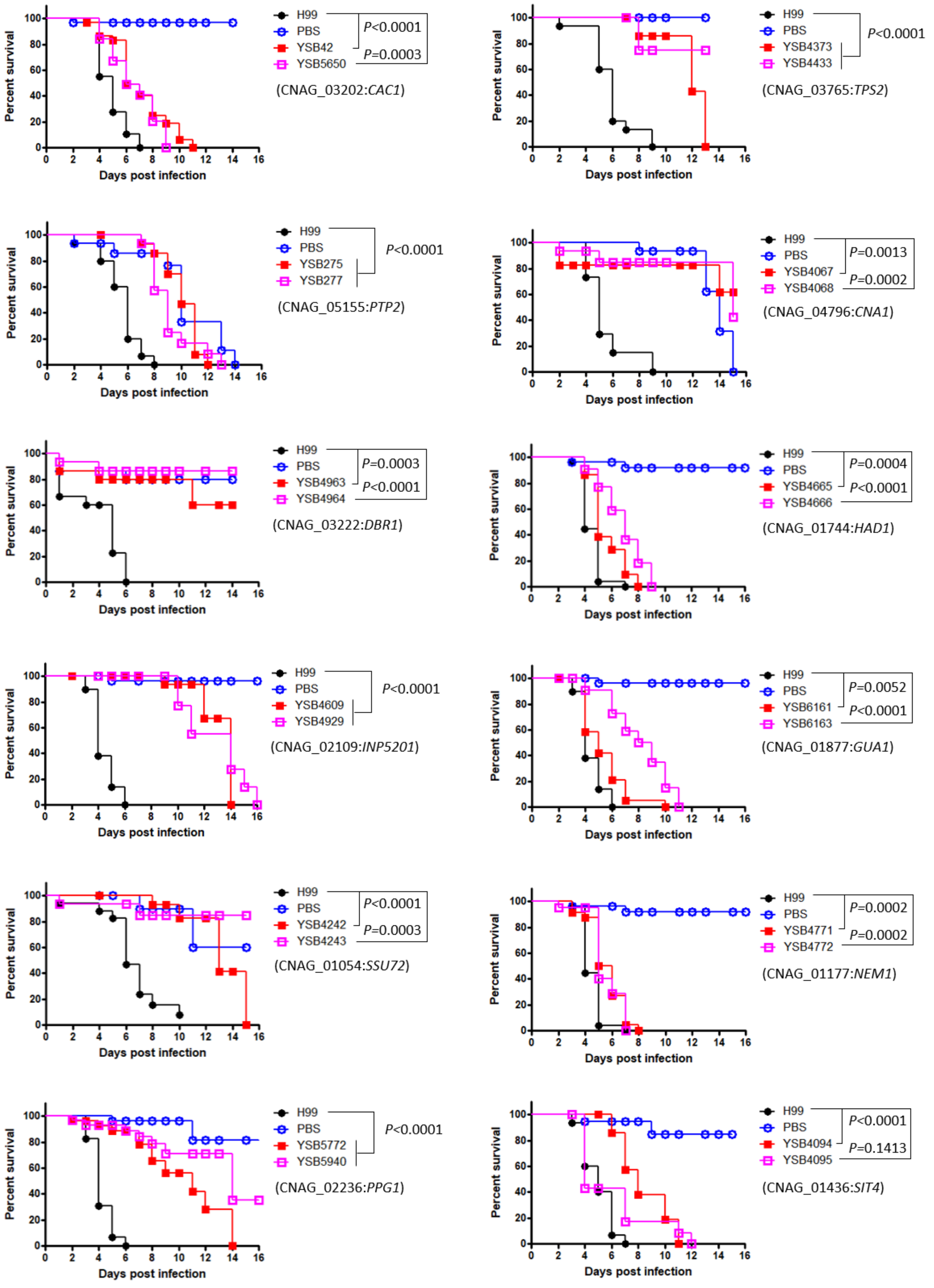
Percent survival
Days post infection
H99
PBS
YSB42
YSB5650
P<0.0001
P=0.0003
(CNAG_03202:CAC1)
H99
PBS
YSB4373
YSB4433
P<0.0001
(CNAG_03765:TPS2)
H99
PBS
YSB275
YSB277
P<0.0001
(CNAG_05155:PTP2)
H99
PBS
YSB4067
YSB4068
P=0.0013
P=0.0002
(CNAG_04796:CNA1)
H99
PBS
YSB4963
YSB4964
P=0.0003
P<0.0001
(CNAG_03222:DBR1)
H99
PBS
YSB4665
YSB4666
P=0.0004
P<0.0001
(CNAG_01744:HAD1)
H99
PBS
YSB4609
YSB4929
P<0.0001
(CNAG_02109:INP5201)
H99
PBS
YSB6161
YSB6163
P=0.0052
P<0.0001
(CNAG_01877:GUA1)
H99
PBS
YSB4242
YSB4243
P<0.0001
P=0.0003
(CNAG_01054:SSU72)
H99
PBS
YSB4771
YSB4772
P=0.0002
P=0.0002
(CNAG_01177:NEM1)
H99
PBS
YSB5772
YSB5940
P<0.0001
(CNAG_02236:PPG1)
H99
PBS
YSB4094
YSB4095
P<0.0001
P=0.1413
(CNAG_01436:SIT4)

Continued

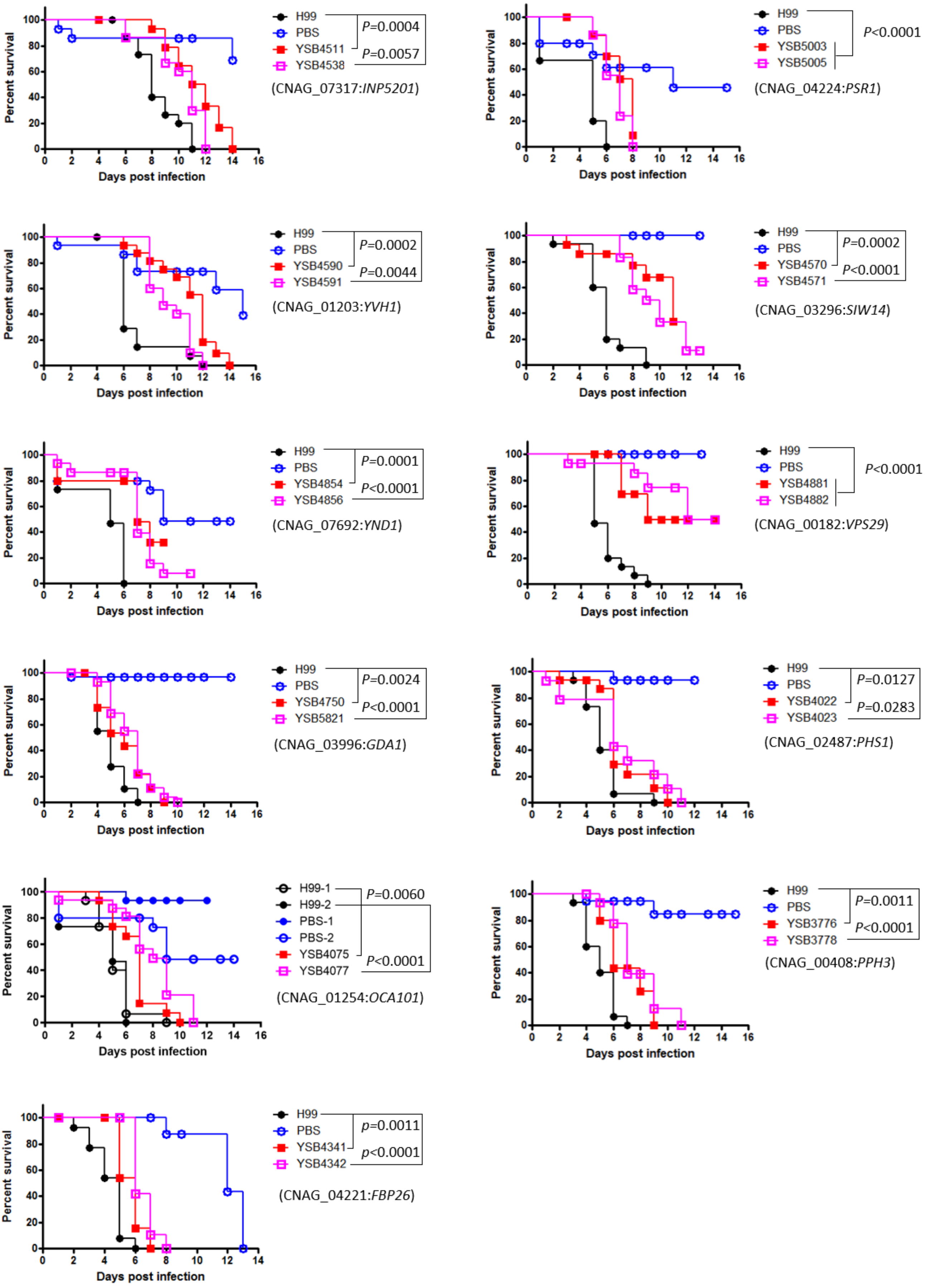
Percent survival
Days post infection
H99
PBS
YSB4511
YSB4538
P=0.0004
P=0.0057
(CNAG_07317:INP5201)
H99
PBS
YSB5003
YSB5005
P<0.0001
(CNAG_04224:PSR1)
H99
PBS
YSB4590
YSB4591
P=0.0002
P=0.0044
(CNAG_01203:YVH1)
H99
PBS
YSB4570
YSB4571
P=0.0002
P<0.0001
(CNAG_03296:SIW14)
H99
PBS
YSB4854
YSB4856
P=0.0001
P<0.0001
(CNAG_07692:YND1)
H99
PBS
YSB4881
YSB4882
P<0.0001
(CNAG_00182:VPS29)
H99
PBS
YSB4750
YSB5821
P=0.0024
P<0.0001
(CNAG_03996:GDA1)
H99
PBS
YSB4022
YSB4023
P=0.0127
P=0.0283
(CNAG_02487:PHS1)
H99-1
H99-2
PBS-1
PBS-2
YSB4075
YSB4077
P=0.0060
P<0.0001
(CNAG_01254:OCA101)
H99
PBS
YSB3776
YSB3778
P=0.0011
P<0.0001
(CNAG_00408:PPH3)
H99
PBS
YSB4341
YSB4342
p=0.0011
p<0.0001
(CNAG_04221:FBP26)

Supplementary Figure 4. Identification of virulence-regulating phosphatases in *C. neoformans* in an insect-killing assay. Each phosphatase mutant was cultured in YPD medium at 30°C for 16 h, pelleted, and washed three times with PBS. Four thousand phosphatase mutant cells were injected into *Galleria mellonella* larvae (at least 15 larvae per group). PBS-only was used as a non-infective negative control, and the H99S strain was used as a positive control. Infected larvae were placed in Petri dishes, incubated in a humidified container at 37°C, and monitored daily. *P* values were calculated using the log-rank (Mantel-Cox) test to measure statistical differences between the wild-type (WT) strain (H99S) and each phosphatase mutant strain. Two independently constructed mutants for each phosphatase gene were monitored.

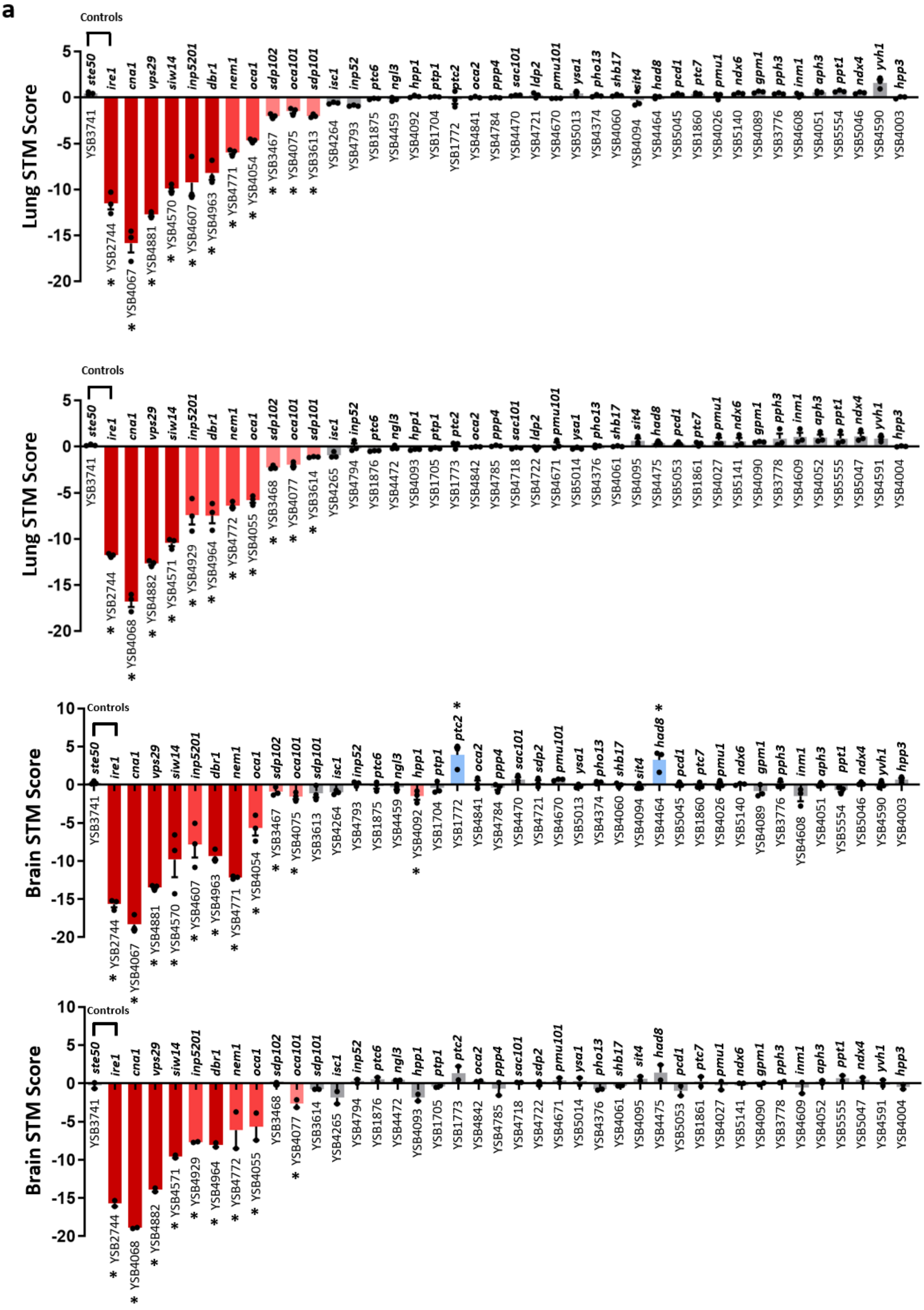
a
Controls
Lung STM Score
Lung STM Score
Brain STM Score
Brain STM Score
ste50 ire1 cna1 vps29 siw14 inp5201 dbr1 nem1 oca1 sdp102 oca101 sdp101 isc1 inp52 ptc6 ngl3 hpp1 ptp1 ptc2 oca2 ppp4 sac101 ldp2 pmu101 ysa1 pho13 shb17 sit4 had8 pcd1 ptc7 pmu1 ndx6 gpm1 pph3 inm1 aph3 ppt1 ndx4 yvh1 hpp3
YSB3741 YSB2744 YSB4067 YSB4881 YSB4570 YSB4607 YSB4963 YSB4771 YSB4054 YSB3467 YSB4075 YSB3613 YSB4264 YSB4793 YSB1875 YSB4459 YSB4092 YSB1704 YSB1772 YSB4841 YSB4784 YSB4470 YSB4721 YSB4670 YSB5013 YSB4374 YSB4060 YSB4094 YSB4464 YSB5045 YSB1860 YSB4026 YSB5140 YSB4089 YSB3776 YSB4608 YSB4051 YSB5554 YSB5046 YSB4590 YSB4003
YSB3741 YSB2744 YSB4068 YSB4882 YSB4571 YSB4929 YSB4964 YSB4772 YSB4055 YSB3468 YSB4077 YSB3614 YSB4265 YSB4794 YSB1876 YSB4472 YSB4093 YSB1705 YSB1773 YSB4842 YSB4785 YSB4718 YSB4722 YSB4671 YSB5014 YSB4376 YSB4061 YSB4095 YSB4475 YSB5053 YSB1861 YSB4027 YSB5141 YSB4090 YSB3778 YSB4609 YSB4052 YSB5555 YSB5047 YSB4591 YSB4004
ste50 ire1 cna1 vps29 siw14 inp5201 dbr1 nem1 oca1 sdp102 oca101 sdp101 isc1 inp52 ptc6 ngl3 hpp1 ptp1 ptc2 oca2 ppp4 sac101 sdp2 pmu101 ysa1 pho13 shb17 sit4 had8 pcd1 ptc7 pmu1 ndx6 gpm1 pph3 inm1 aph3 ppt1 ndx4 yvh1 hpp3
5 0 -5 -10 -15 -20
10 5 0 -5 -10 -15 -20
*

Continued

b

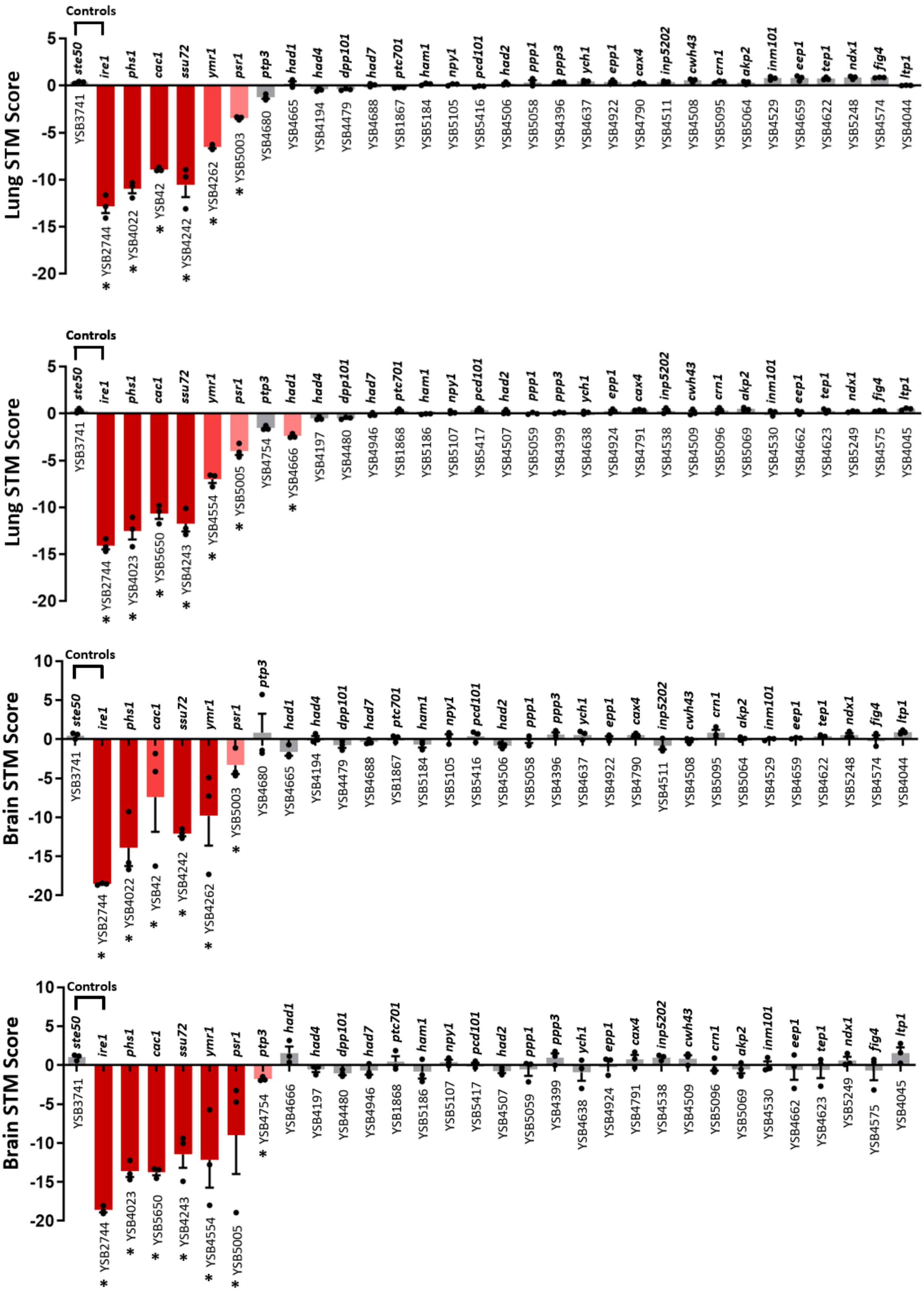

Controls
Lung STM Score
5
0
-5
-10
-15
-20
ste50 ire1 phs1 cac1 ssu72 ymr1 psr1 ptp3 had1 had4 dpp101 had7 ptc701 ham1 npy1 pcd101 had2 ppp1 ppp3 ych1 epp1 cax4 inp5202 cwh43 crn1 akp2 inm101 eep1 tep1 ndx1 fig4 ltp1
YSB3741 YSB2744 YSB4022 YSB42 YSB4242 YSB4262 YSB5003 YSB4680 YSB4665 YSB4194 YSB4479 YSB4688 YSB1867 YSB5184 YSB5105 YSB5416 YSB4506 YSB5058 YSB4396 YSB4637 YSB4922 YSB4790 YSB4511 YSB4508 YSB5095 YSB5064 YSB4529 YSB4659 YSB4622 YSB5248 YSB4574 YSB4044
Controls
Lung STM Score
5
0
-5
-10
-15
-20
ste50 ire1 phs1 cac1 ssu72 ymr1 psr1 ptp3 had1 had4 dpp101 had7 ptc701 ham1 npy1 pcd101 had2 ppp1 ppp3 ych1 epp1 cax4 inp5202 cwh43 crn1 akp2 inm101 eep1 tep1 ndx1 fig4 ltp1
YSB3741 YSB2744 YSB4023 YSB5650 YSB4243 YSB4554 YSB5005 YSB4754 YSB4666 YSB4197 YSB4480 YSB4946 YSB1868 YSB5186 YSB5107 YSB5417 YSB4507 YSB5059 YSB4399 YSB4638 YSB4924 YSB4791 YSB4538 YSB4509 YSB5096 YSB5069 YSB4530 YSB4662 YSB4623 YSB5249 YSB4575 YSB4045
Controls
Brain STM Score
10
5
0
-5
-10
-15
-20
ste50 ire1 phs1 cac1 ssu72 ymr1 psr1 ptp3 had1 had4 dpp101 had7 ptc701 ham1 npy1 pcd101 had2 ppp1 ppp3 ych1 epp1 cax4 inp5202 cwh43 crn1 akp2 inm101 eep1 tep1 ndx1 fig4 ltp1
YSB3741 YSB2744 YSB4022 YSB42 YSB4242 YSB4262 YSB5003 YSB4680 YSB4665 YSB4194 YSB4479 YSB4688 YSB1867 YSB5184 YSB5105 YSB5416 YSB4506 YSB5058 YSB4396 YSB4637 YSB4922 YSB4790 YSB4511 YSB4508 YSB5095 YSB5064 YSB4529 YSB4659 YSB4622 YSB5248 YSB4574 YSB4044
Controls
Brain STM Score
10
5
0
-5
-10
-15
-20
ste50 ire1 phs1 cac1 ssu72 ymr1 psr1 ptp3 had1 had4 dpp101 had7 ptc701 ham1 npy1 pcd101 had2 ppp1 ppp3 ych1 epp1 cax4 inp5202 cwh43 crn1 akp2 inm101 eep1 tep1 ndx1 fig4 ltp1
YSB3741 YSB2744 YSB4023 YSB5650 YSB4243 YSB4554 YSB5005 YSB4754 YSB4666 YSB4197 YSB4480 YSB4946 YSB1868 YSB5186 YSB5107 YSB5417 YSB4507 YSB5059 YSB4399 YSB4638 YSB4924 YSB4791 YSB4538 YSB4509 YSB5096 YSB5069 YSB4530 YSB4662 YSB4623 YSB5249 YSB4575 YSB4045

Continued

c

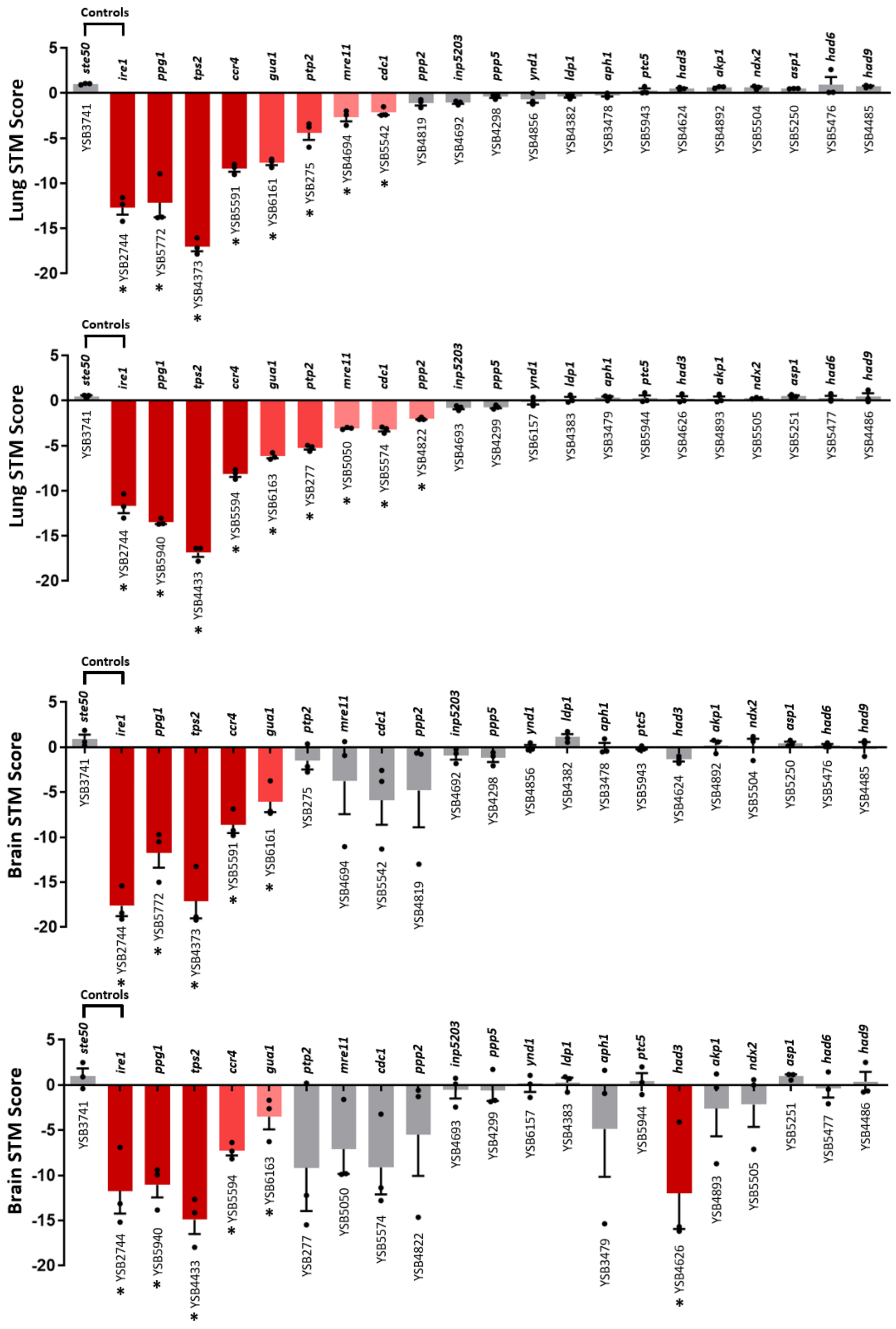
Controls
Lung STM Score
5
0
-5
-10
-15
-20
ste50 ire1 ppg1 tps2 ccr4 gua1 ptp2 mre11 cdc1 ppp2 inp5203 ppp5 ynd1 ldp1 aph1 ptc5 had3 akp1 ndx2 asp1 had6 had9
YSB3741 * YSB2744 * YSB5772 * YSB4373 * YSB5591 * YSB6161 * YSB275 * YSB4694 * YSB5542 YSB4819 YSB4692 YSB4298 YSB4856 YSB4382 YSB3478 YSB5943 YSB4624 YSB4892 YSB5504 YSB5250 YSB5476 YSB4485
Controls
Lung STM Score
ste50 ire1 ppg1 tps2 ccr4 gua1 ptp2 mre11 cdc1 ppp2 inp5203 ppp5 ynd1 ldp1 aph1 ptc5 had3 akp1 ndx2 asp1 had6 had9
YSB3741 * YSB2744 * YSB5940 * YSB4433 * YSB5594 * YSB6163 * YSB277 * YSB5050 * YSB5574 * YSB4822 YSB4693 YSB4299 YSB6157 YSB4383 YSB3479 YSB5944 YSB4626 YSB4893 YSB5505 YSB5251 YSB5477 YSB4486
Controls
Brain STM Score
ste50 ire1 ppg1 tps2 ccr4 gua1 ptp2 mre11 cdc1 ppp2 inp5203 ppp5 ynd1 ldp1 aph1 ptc5 had3 akp1 ndx2 asp1 had6 had9
YSB3741 * YSB2744 * YSB5772 * YSB4373 * YSB5591 * YSB6161 YSB275 YSB4694 YSB5542 YSB4819 YSB4692 YSB4298 YSB4856 YSB4382 YSB3478 YSB5943 YSB4624 YSB4892 YSB5504 YSB5250 YSB5476 YSB4485
Controls
Brain STM Score
ste50 ire1 ppg1 tps2 ccr4 gua1 ptp2 mre11 cdc1 ppp2 inp5203 ppp5 ynd1 ldp1 aph1 ptc5 had3 akp1 ndx2 asp1 had6 had9
YSB3741 * YSB2744 * YSB5940 * YSB4433 * YSB5594 * YSB6163 YSB277 YSB5050 YSB5574 YSB4822 YSB4693 YSB4299 YSB6157 YSB4383 YSB3479 YSB5944 * YSB4626 YSB4893 YSB5505 YSB5251 YSB5477 YSB4486

Continued

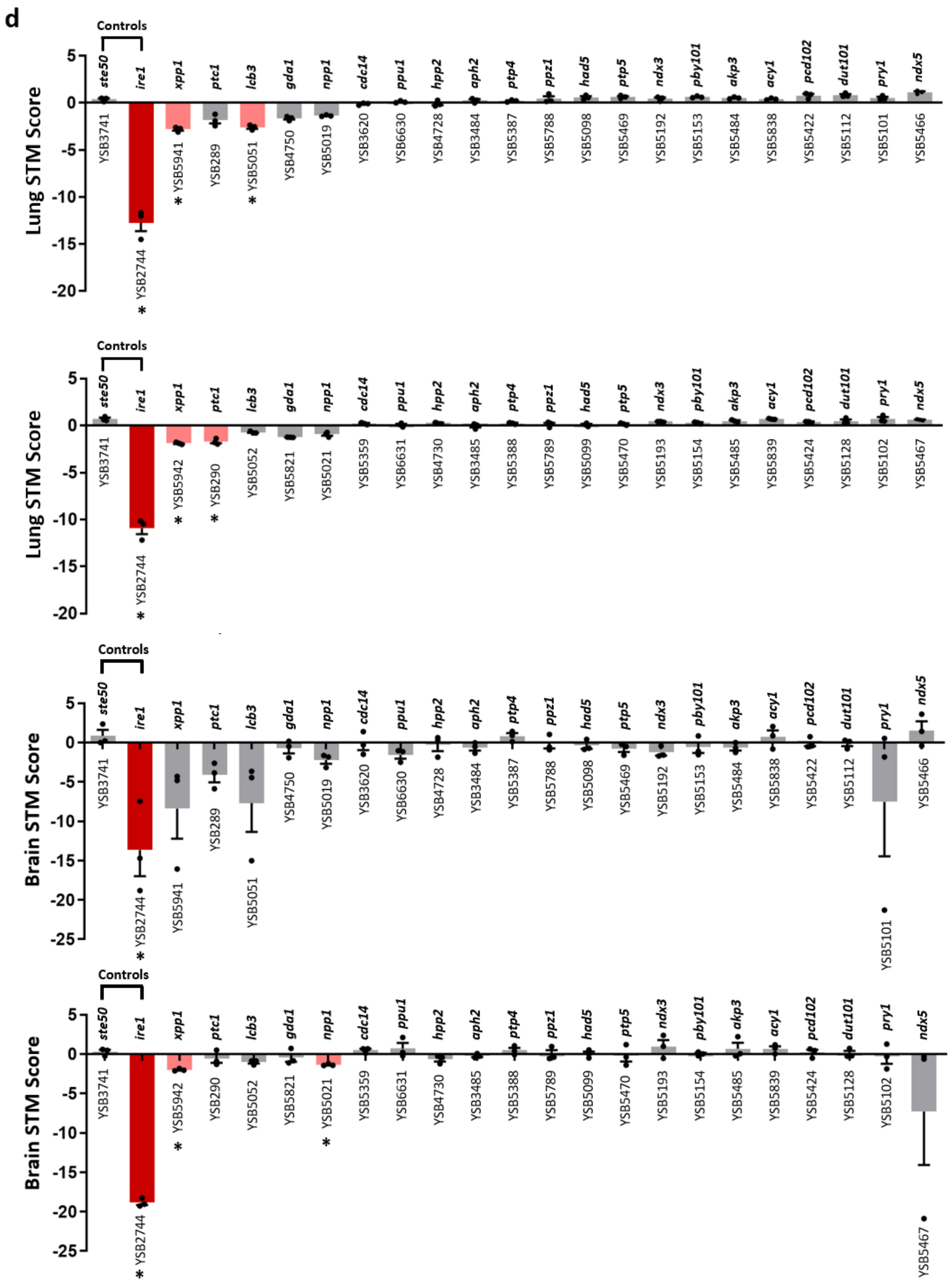
d
Controls
Lung STM Score
Brain STM Score
ste50
ire1
xpp1
ptc1
lcb3
gda1
npp1
cdc14
ppu1
hpp2
aph2
ptp4
ppz1
had5
ptp5
ndx3
pby101
akp3
acy1
pcd102
dut101
pry1
ndx5

Continued

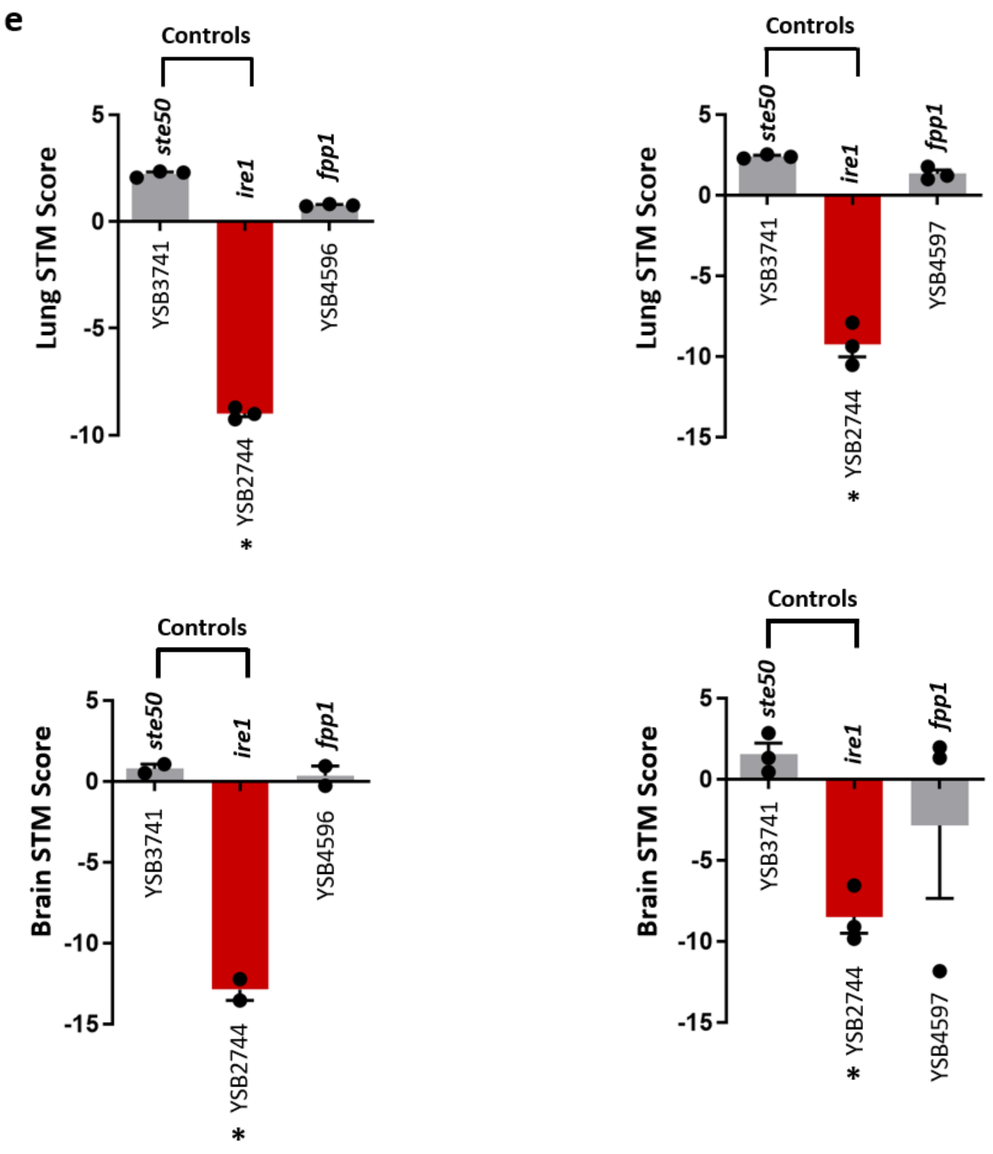

Supplementary Figure 5. Identification of infectivity-regulating phosphatases in *C. neoformans* in a signature-tagged mutagenesis (STM)-based murine infectivity assay. Lung and brain STM scores were calculated by quantitative PCR using a common primer and the signature tag-specific primers listed in Supplementary Data 4. The *ste50*Δ and *ire*Δ mutants were used as virulent positive control and avirulent negative control strains, respectively, as previously reported[3,4]. (**a**-**e**) Phosphatase mutants were divided into five sets. Each panel indicates a separate pooled set of mutants. Each set included two independent strains of each phosphatase mutant. The statistically significant was calculated by one-way ANOVA analysis with Bonferroni's multiple comparison test. Data are presented as mean values ± standard error of mean (SEM) (*, P<0.05) and presented with different colour codes: pink (<−2), reddish (<−4), red (<−8), and light blue (>2).

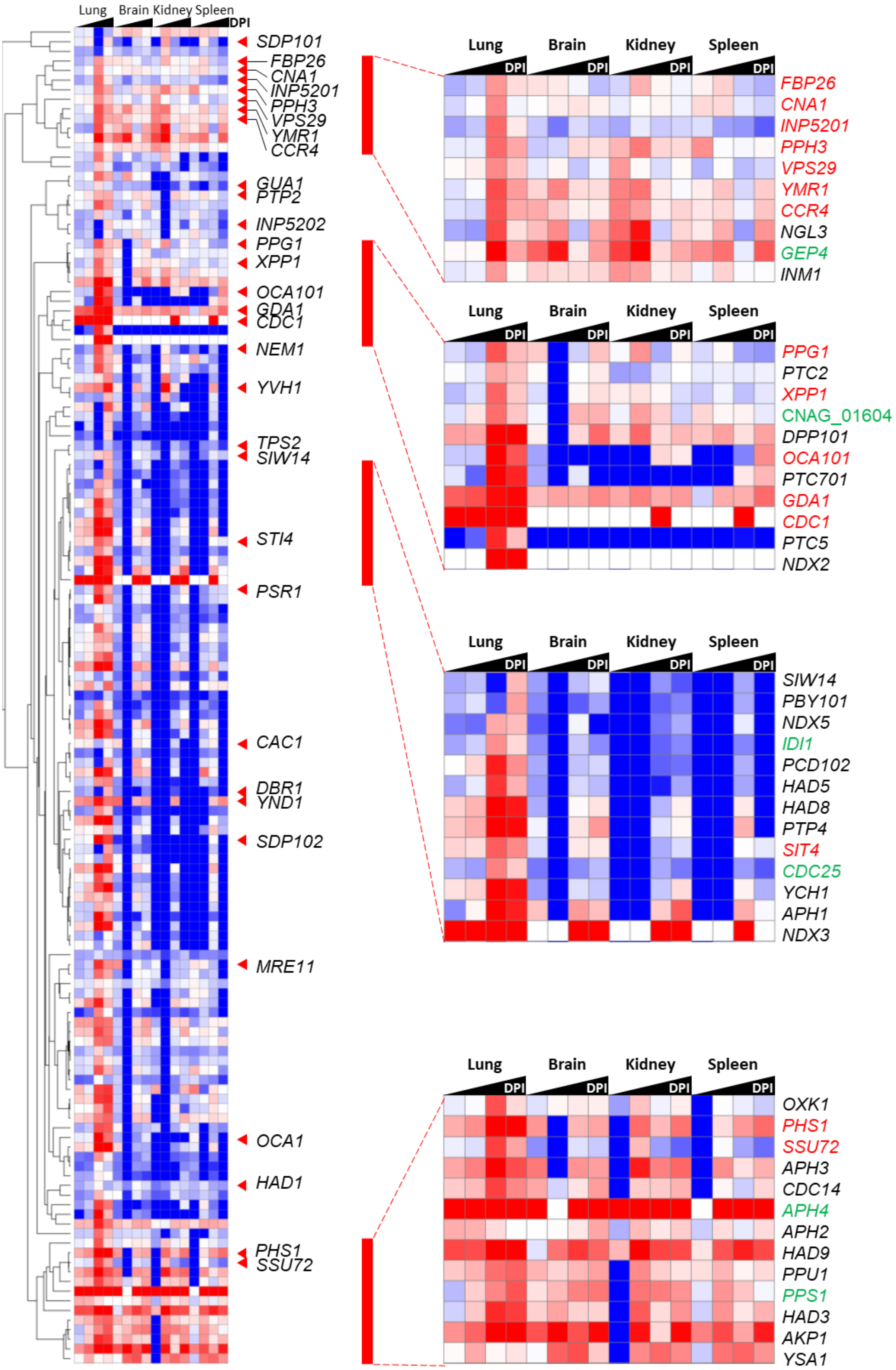
Lung Brain Kidney Spleen
DPI
SDP101
FBP26
CNA1
INP5201
PPH3
VPS29
YMR1
CCR4
GUA1
PTP2
INP5202
PPG1
XPP1
OCA101
GDA1
CDC1
NEM1
YVH1
TPS2
SIW14
STI4
PSR1
CAC1
DBR1
YND1
SDP102
MRE11
OCA1
HAD1
PHS1
SSU72
Lung
Brain
Kidney
Spleen
DPI
FBP26
CNA1
INP5201
PPH3
VPS29
YMR1
CCR4
NGL3
GEP4
INM1
PPG1
PTC2
XPP1
CNAG_01604
DPP101
OCA101
PTC701
GDA1
CDC1
PTC5
NDX2
SIW14
PBY101
NDX5
IDI1
PCD102
HAD5
HAD8
PTP4
SIT4
CDC25
YCH1
APH1
NDX3
OXK1
PHS1
SSU72
APH3
CDC14
APH4
APH2
HAD9
PPU1
PPS1
HAD3
AKP1
YSA1

Supplementary Figure 6. Clustering of *in vivo* gene expression data of *Cryptococcus neoformans* phosphatases. *In vivo* gene expression profiles of *C. neoformans* phosphatases were hierarchically clustered using one minus Pearson correlation in Morpheus (https://software.broadinstitute.org/morpheus). The original *in vivo* gene expression data obtained from the NanoString nCounter analysis are available in Supplementary Data 8. Of the 139 phosphatases, the pathogenicity-related phosphatases are given in the left panel. The groups highlighted in the right panel include the phosphatase genes that exhibited high *in vivo* expression in the lungs, brain, and/or kidney. Red letters indicate the pathogenicity-related phosphatases and green letters indicate putative essential phosphatases in *C. neoformans*.

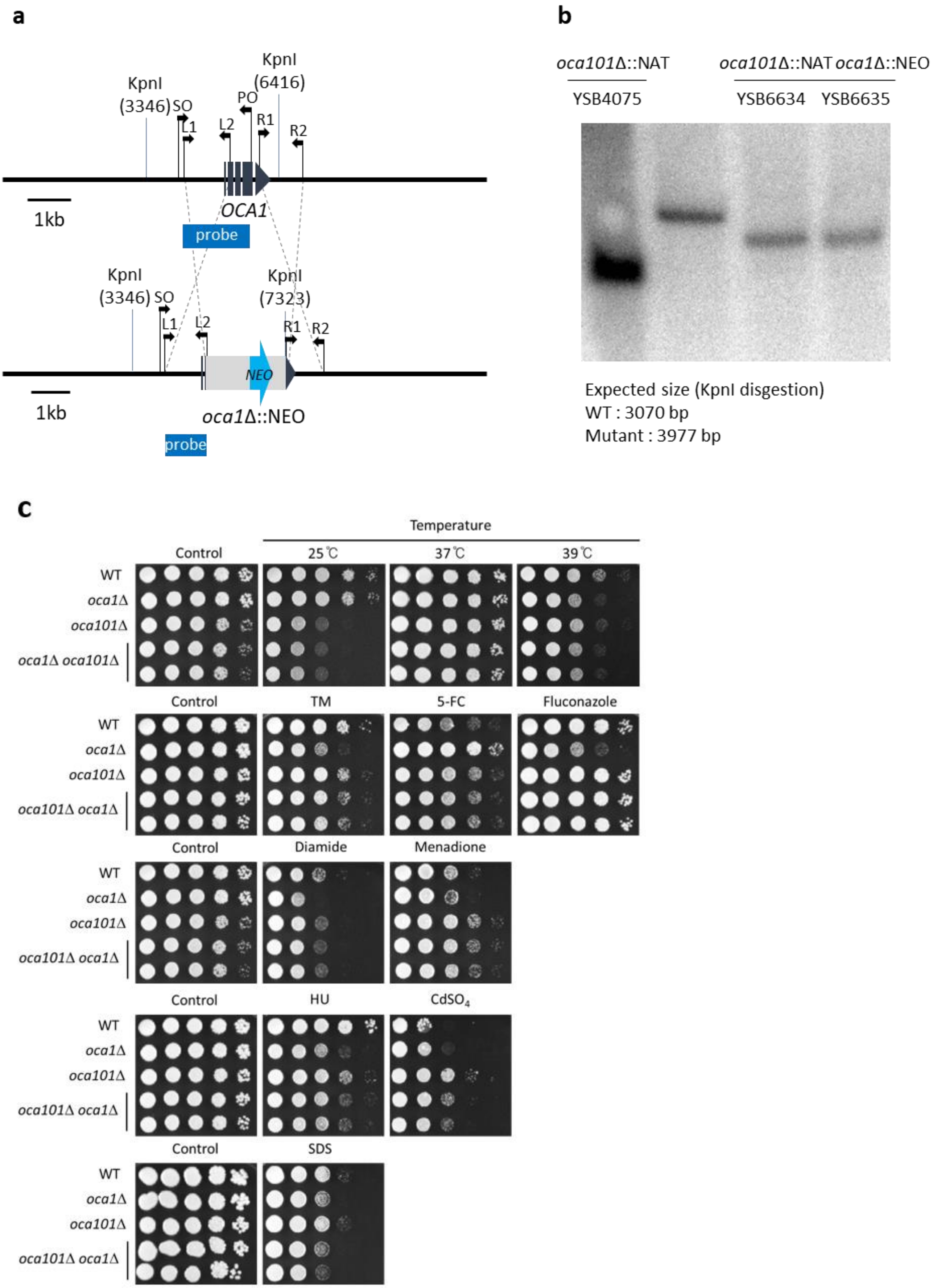
a
KpnI
(3346)
SO
L1
L2
PO
R1
R2
KpnI
(6416)
1kb
OCA1
probe
KpnI
(3346)
SO
L1
L2
KpnI
(7323)
R1
R2
NEO
1kb
oca1Δ::NEO
probe
b
oca101Δ::NAT
YSB4075
oca101Δ::NAT oca1Δ::NEO
YSB6634
YSB6635
Expected size (KpnI disgestion)
WT : 3070 bp
Mutant : 3977 bp
c
Temperature
Control
25℃
37℃
39℃
WT
oca1Δ
oca101Δ
oca1Δ oca101Δ
Control
TM
5-FC
Fluconazole
WT
oca1Δ
oca101Δ
oca101Δ oca1Δ
Control
Diamide
Menadione
WT
oca1Δ
oca101Δ
oca101Δ oca1Δ
Control
HU
CdSO4
WT
oca1Δ
oca101Δ
oca101Δ oca1Δ
Control
SDS
WT
oca1Δ
oca101Δ
oca101Δ oca1Δ

Supplementary Figure 7. ***In vitro*** **phenotypic traits of the** ***oca1*****Δ**, ***oca101*****Δ**, and ***oca1*****Δ** ***oca101*****Δ** **mutants.** (**a**) Strategy for disruption of the *OCA1* gene in *oca101*Δ mutant strain (YSB4075). (**b**) Genotype of *oca1*Δ *oca101*Δ mutant strains were confirmed by Southern blot analysis using genomic DNA digested with the restriction enzyme KpnI. The Southern blot analysis was repeated twice and one representative image was shown here. Source data are provided as a Source Data file. (**c**) Wild-type (WT; H99S), *oca1*Δ, *oca101*Δ, and *oca1*Δ *oca101*Δ mutant strains grown for 16 h at 30°C in liquid YPD medium, serially diluted 10-fold (1 to 10^4), and spotted on YPD agar media containing the indicated concentrations of the following chemical agents: 0.3 µg per ml tunicamycin (TM), 500 µg per ml flucytosine (5-FC), 13 µg per ml fluconazole (FCZ), 2.5 mM diamide, 0.02 mM menadione, 110 mM hydroxyl urea (HU), 25 µm cadmium sulphate ($CdSO_4$), or 0.03% sodium dodecyl sulphate (SDS). Cells were incubated at 30°C or indicated temperature and photographed after 2–3 days. This is the representative of more than three biologically independent experiments.

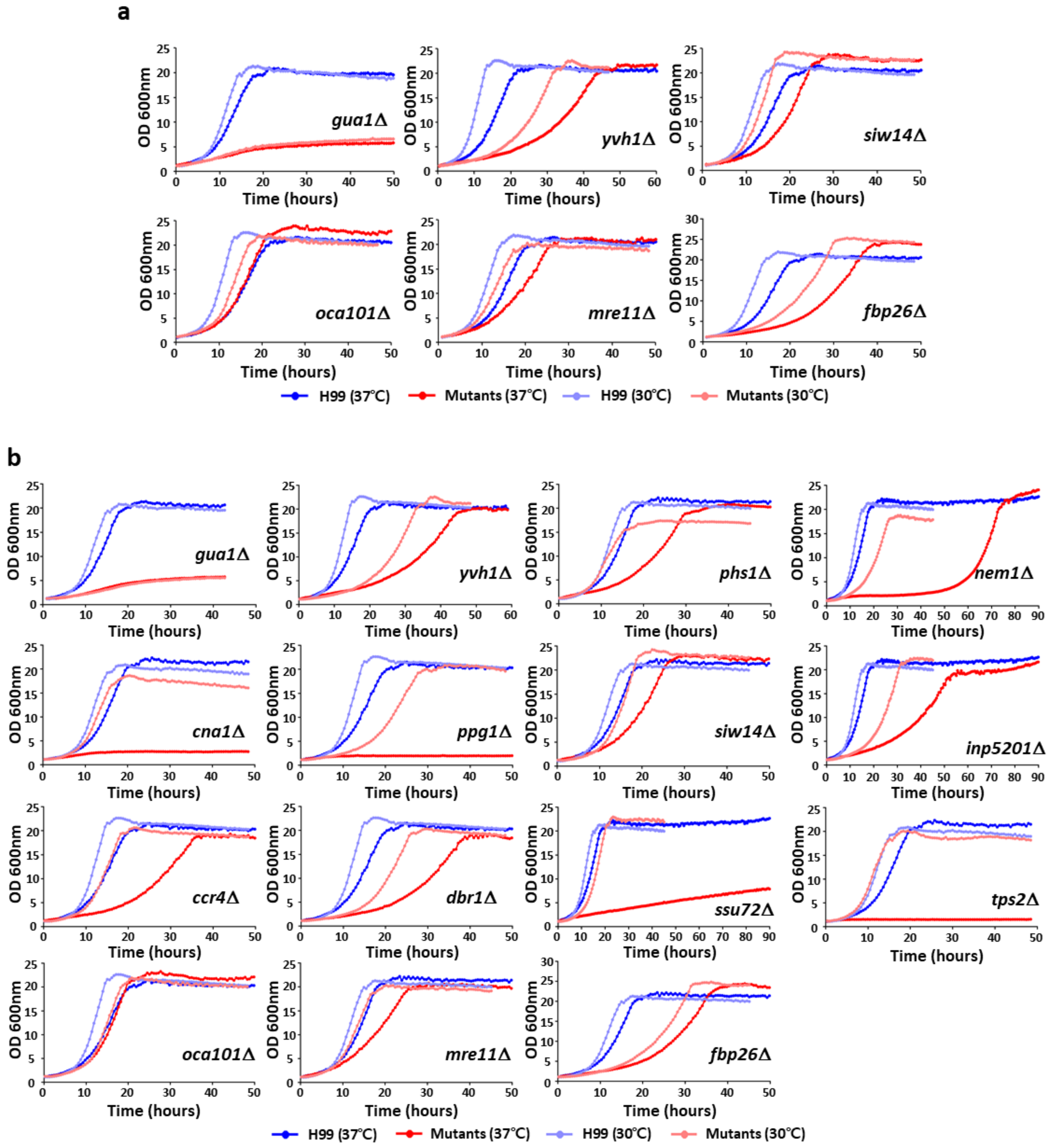

Supplementary Figure 8. Growth curves of phosphatase mutants defective in growth at mammalian body temperature. (**a-b**) and Figure 4a show the growth rates of the temperature sensitive phosphatase mutants from two biologically independent experiments. *Cryptococcus neoformans* wild-type (WT) strain (H99S) and phosphatase mutants were cultured at 30°C for 16 h and then inoculated into fresh liquid YPD medium (OD_{600nm} = 0.2). Cells were than incubated at 30°C or 37°C in a multi-channel bioreactor (Biosan Laboratories, Inc., Warren, MI), and OD_{600nm} was automatically measured for 40 to 90 h.

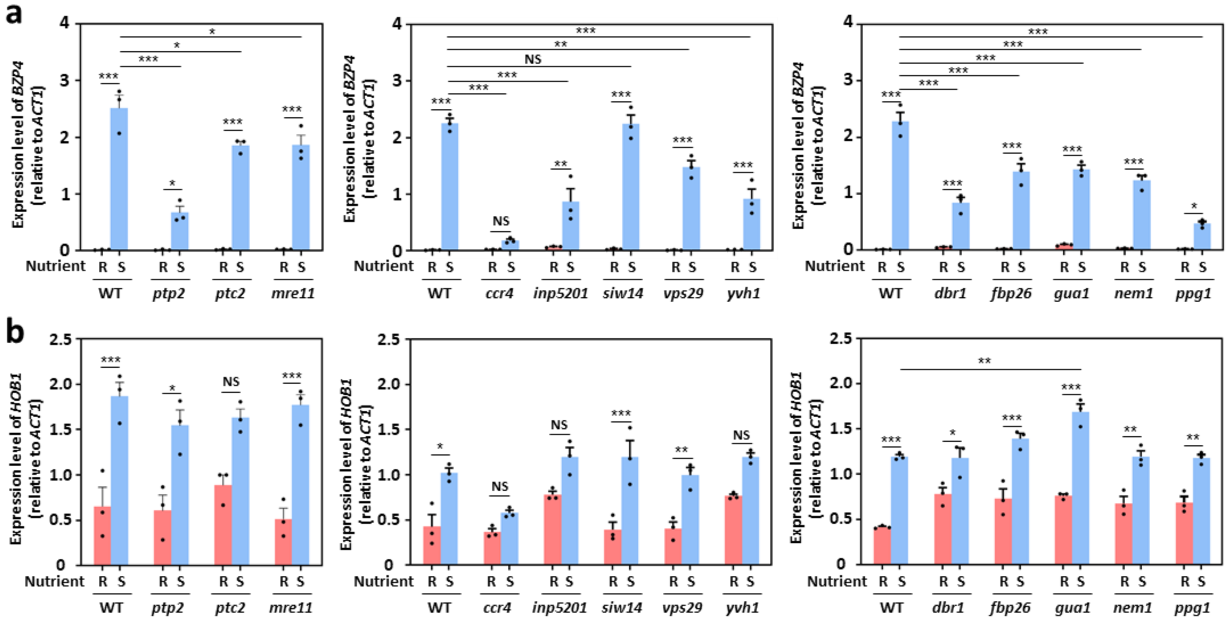

Supplementary Figure 9. Expression levels of known melanin-regulating genes in phosphatase mutants. (**a-b**) Gene expression (qRT-PCR) of melanin-regulating genes *BZP4*, and *HOB1* in both nutrient-rich (R) and nutrient-starvation (S) conditions. RNA was extracted from three biological replicates with three technical replicates of wild-type and melanin-regulating phosphatase mutants; expression was normalised to *ACT1*. Statistical significance was calculated by one-way ANOVA analysis with Bonferroni's multiple comparison test. Data are presented as mean values ± standard error of mean (SEM) (*, P<0.05; **, P<0.001; ***, P<0.0001).

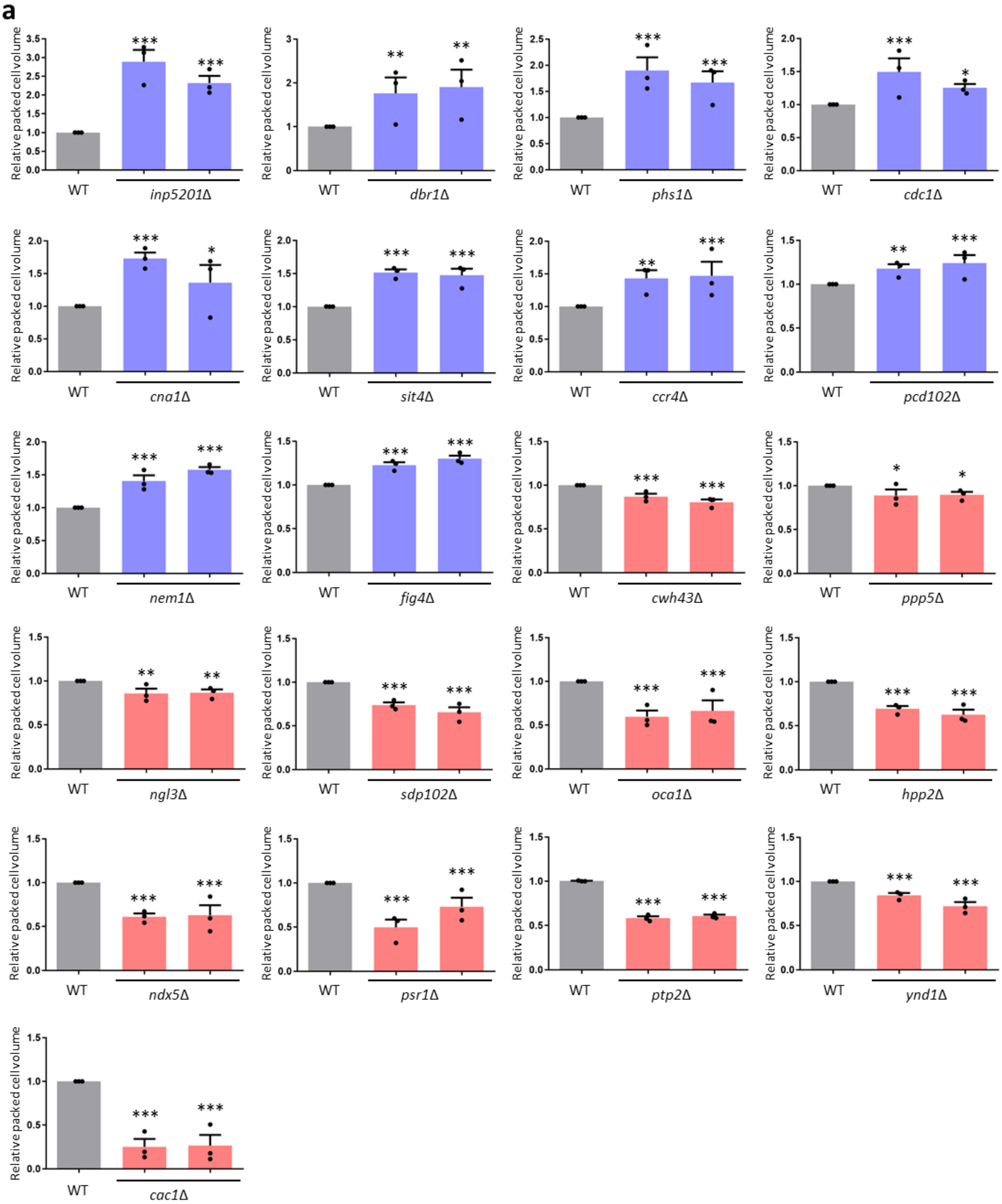
a
Relative packed cell volume
WT
inp5201Δ
dbr1Δ
phs1Δ
cdc1Δ
cna1Δ
sit4Δ
ccr4Δ
pcd102Δ
nem1Δ
fig4Δ
cwh43Δ
ppp5Δ
ngl3Δ
sdp102Δ
oca1Δ
hpp2Δ
ndx5Δ
psr1Δ
ptp2Δ
ynd1Δ
cac1Δ

Continued

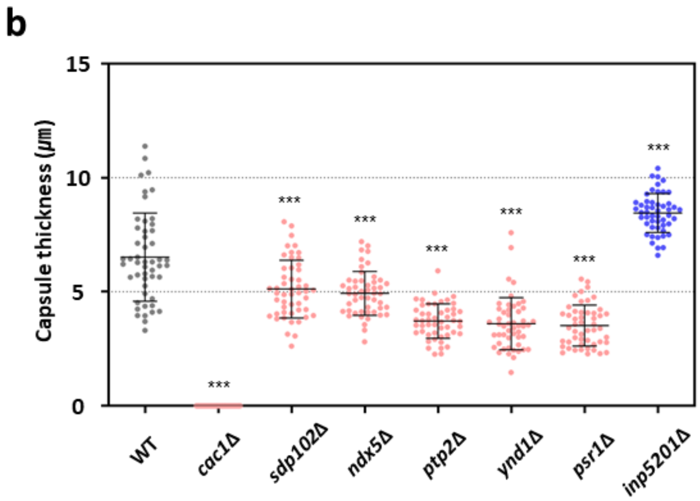

Supplementary Figure 10. Capsule-regulating phosphatases in *C. neoformans*. (**a**) Each phosphatase mutant was cultured for 16 h at 30°C, spotted onto Dulbecco's Modified Eagle's (DME) agar medium, and incubated at 37°C for 2 days to induce capsule production. Cells were then scraped and fixed by 10% formalin. Fixed cells were adjusted to 3×10^8 cells per ml, and 50 µl of the cell suspension was injected into microhaematocrit capillary tubes (Kimble Chase, Rockwood, TN). Packed cell volume ratio (packed cell phase / total phase) was measured and the relative packed cell volume of each mutant was calculated by normalizing each ratio with packed cell volume ratio of wild-type (WT) H99S strain. Statistical differences in relative packed cell volume ratios were determined by one-way ANOVA analysis with Bonferroni's multiple comparison test. Data are presented as mean values ± standard error of mean (SEM) (*, $P<0.05$; **, $P<0.001$; ***, $P<0.0001$). Each data plot indicates the three biologically independent experiments with three technical replicates. (**b**) Capsule production assay was performed using capsule-inducing media (Littman's agar medium). To measure capsule thickness (total diameter – cell body diameter), 50 cells were measured from the wild-type (WT) strain and each phosphatase mutant (n=50). Statistical significance was calculated by one-way ANOVA analysis with Bonferroni's multiple comparison test. Data are presented as mean values ± standard deviation (*, $P<0.05$; **, $P<0.001$; ***, $P<0.0001$). This graph is representative of more than three independent experiments.

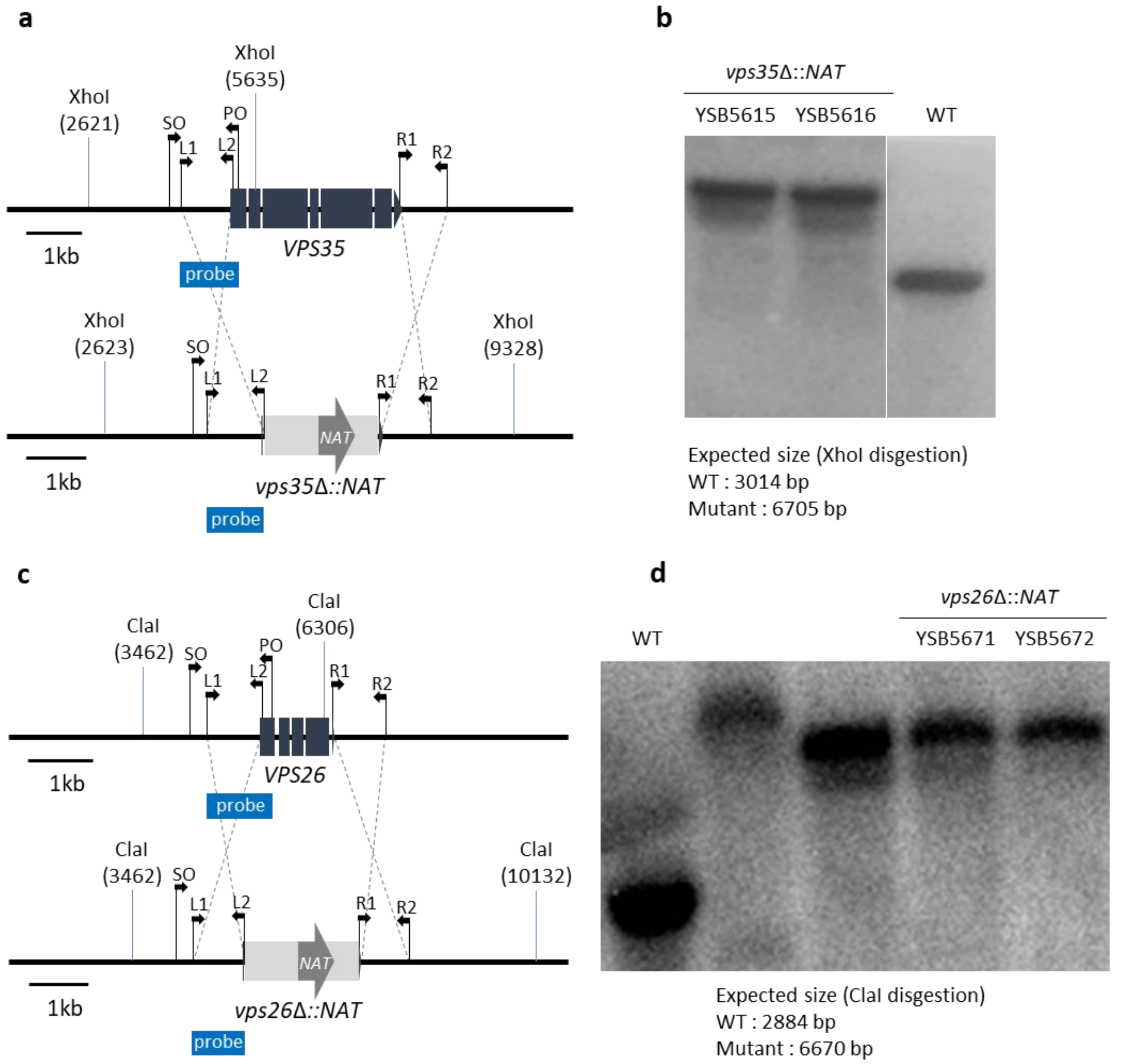

a
XhoI
(5635)
XhoI
(2621)
SO
PO
L1
L2
R1
R2
VPS35
1kb
probe
XhoI
(2623)
SO
L1
L2
R1
R2
XhoI
(9328)
NAT
1kb
vps35Δ::NAT
probe
b
vps35Δ::NAT
YSB5615
YSB5616
WT
Expected size (XhoI disgestion)
WT : 3014 bp
Mutant : 6705 bp
c
ClaI
(6306)
ClaI
(3462)
SO
PO
L1
L2
R1
R2
VPS26
1kb
probe
ClaI
(3462)
SO
L1
L2
R1
R2
ClaI
(10132)
NAT
1kb
vps26Δ::NAT
probe
d
vps26Δ::NAT
WT
YSB5671
YSB5672
Expected size (ClaI disgestion)
WT : 2884 bp
Mutant : 6670 bp

Continued

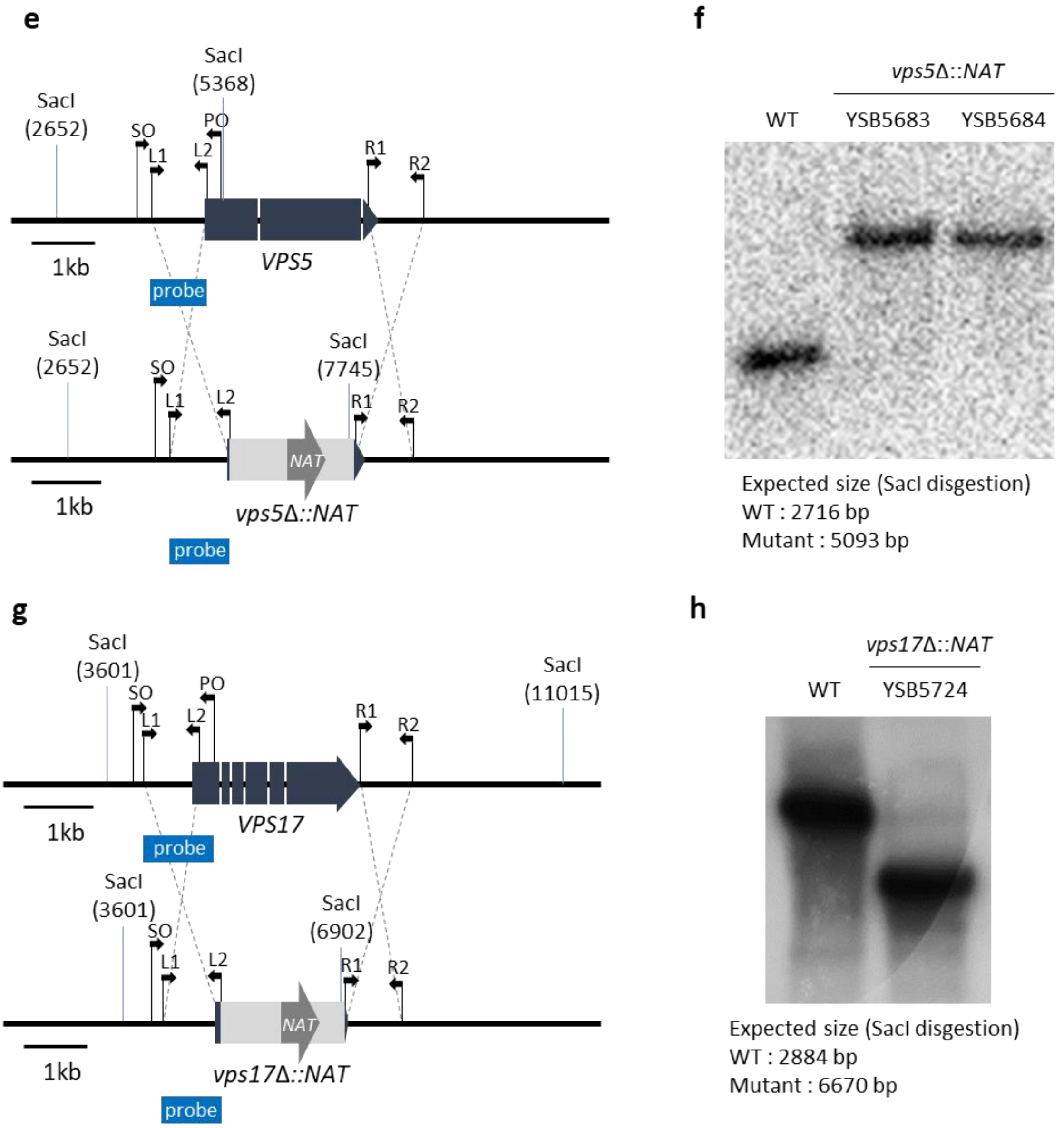

Supplementary Figure 11. Construction of retromer mutant strains. (**a**) Strategy for disruption of the *VPS35* gene in *C. neoformans* H99S. (**b**) Genotype of *vps35*Δ mutant strains were confirmed by Southern blot analysis using genomic DNA digested with the restriction enzyme XhoI. The Southern blot analysis was repeated twice and one representative image was shown here. (**c**) Strategy for disruption of the *VPS26* gene in *C. neoformans* H99S. (**d**) Genotype of *vps26*Δ mutant strains were confirmed by Southern blot analysis using genomic DNA digested with the restriction enzyme ClaI. The Southern blot analysis was repeated twice and one representative image was shown here. (**e**) Strategy for disruption of the *VPS5* gene in *C. neoformans* H99S. (**f**) Genotype of *vps5*Δ mutant strains were confirmed by Southern blot analysis using genomic DNA digested with the restriction enzyme SacI. The Southern blot analysis was repeated twice and one representative image was shown here. (**g**) Strategy for disruption of the *VPS17* gene in *C. neoformans* H99S. (**h**) Genotype of *vps17*Δ mutant strain was confirmed by Southern blot analysis using genomic DNA digested with the restriction enzyme SacI. The Southern blot analysis was repeated twice and one representative image was shown here. Source data are provided as a Source Data file.

a

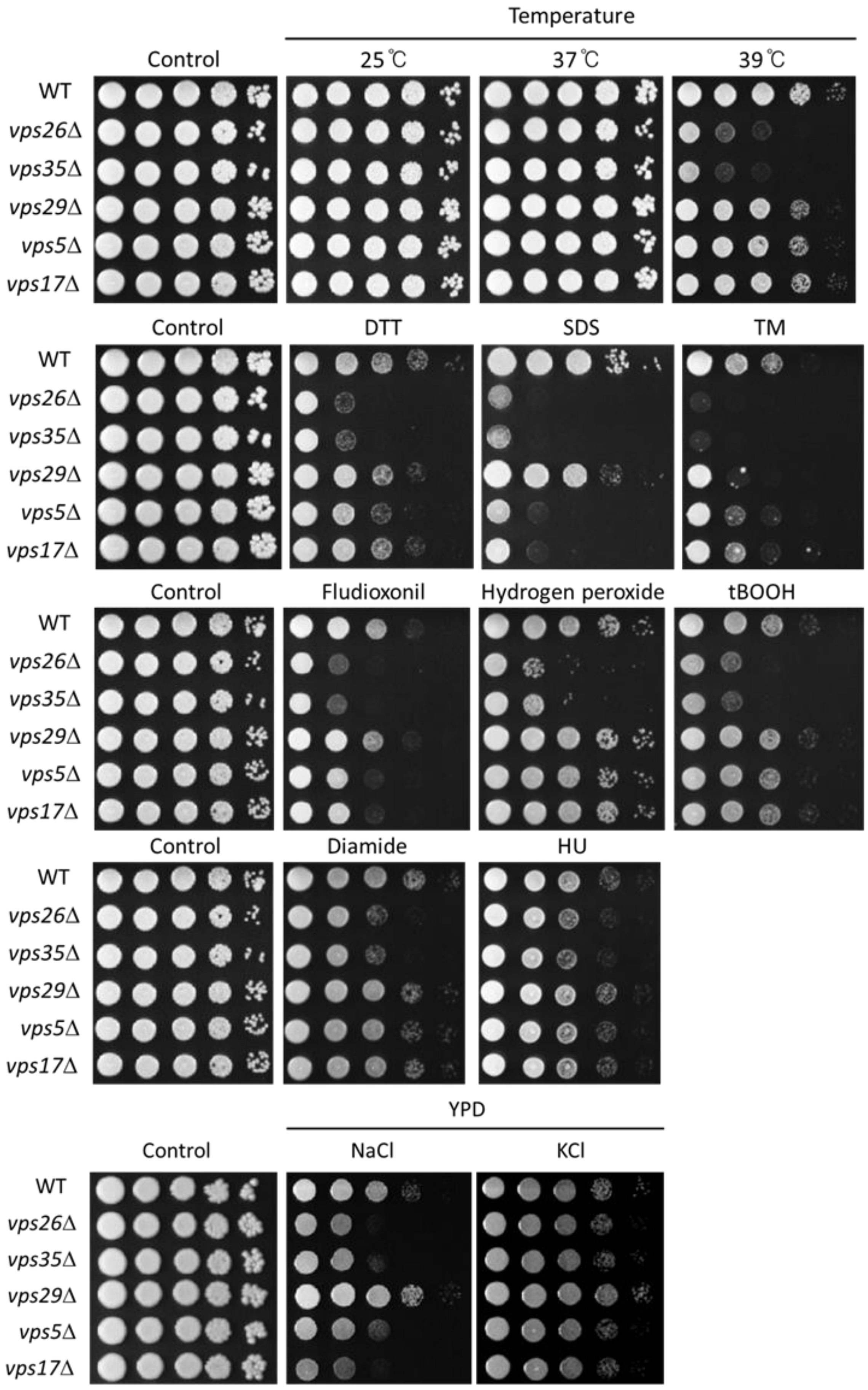
Temperature
Control
25℃
37℃
39℃
WT
vps26Δ
vps35Δ
vps29Δ
vps5Δ
vps17Δ
Control
DTT
SDS
TM
WT
vps26Δ
vps35Δ
vps29Δ
vps5Δ
vps17Δ
Control
Fludioxonil
Hydrogen peroxide
tBOOH
WT
vps26Δ
vps35Δ
vps29Δ
vps5Δ
vps17Δ
Control
Diamide
HU
WT
vps26Δ
vps35Δ
vps29Δ
vps5Δ
vps17Δ
YPD
Control
NaCl
KCl
WT
vps26Δ
vps35Δ
vps29Δ
vps5Δ
vps17Δ

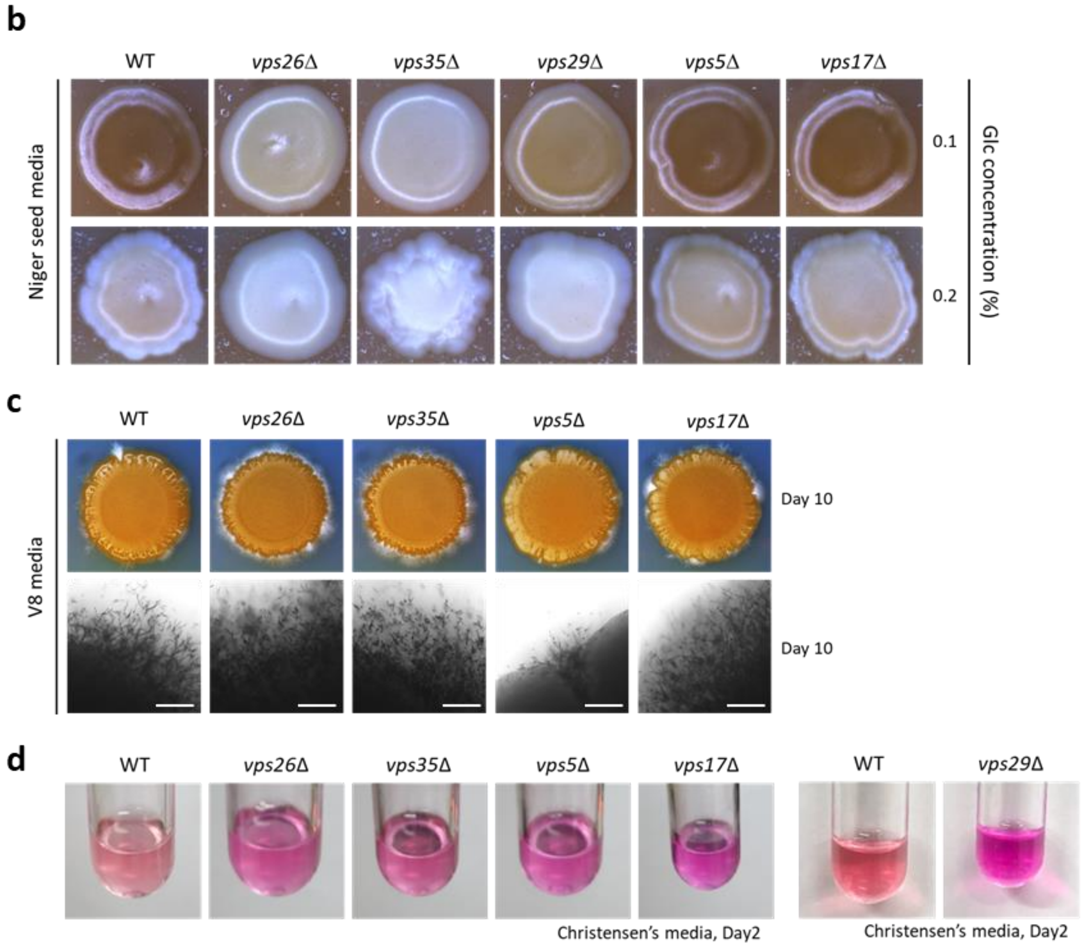

Supplementary Figure 12. *In vitro* phenotypic traits of retromer complex. (**a**-**d**) All data were representative of more than three biologically independent experiments. (**a**) Wild-type (WT) H99S strain and *vps29*Δ, *vps26*Δ, *vps35*Δ, *vps5*Δ, and *vps17*Δ mutants were grown for 16 h at 30°C in liquid YPD medium, serially diluted 10-fold (1 to 10^4), and spotted on YPD agar media containing the indicated concentration of the following chemical agents: 0.3 µg per ml tunicamycin (TM), 500 µg per ml flucytosine (5-FC), 13 µg per ml fluconazole (FCZ), 2.5 mM diamide, 0.02 mM menadione, 110 mM hydroxyl urea (HU), 25 µm cadmium sulphate ($CdSO_4$), 0.03% sodium dodecyl sulphate (SDS), 1 µg per ml fludioxonil, 3.5 mM hydrogen peroxide, 0.7 mM *tert*-butyl hydroperoxide (tBOOH), 1.5 M KCl (1 M KCl in YP medium), or 1.5 M NaCl (1 M NaCl in YP medium). Cells were incubated at 30°C or the indicated temperature and photographed. (**b**) Retromer mutant strains were grown for 16 h at 30°C in liquid YPD medium and washed with PBS. Each strain was spotted on Niger seed media containing 0.1% or 0.2%. glucose, incubated at 37°C, and photographed after 1–4 days. (**c**) Retromer mutant strains and the *MAT***a** (KN99) strain were cultured in YPD medium for 16 h at 30°C, washed twice with phosphate-buffered saline (PBS), mixed at equal concentrations (10^7 cells per ml), spotted on V8 mating media (pH 5), and incubated at room temperature in the dark for 7 to 14 days. Filamentous growth was observed and photographed weekly. Scale bars, 0.25 mm. (**d**) Retromer mutant strains were cultured at 30°C for 16 h, washed with PBS, and an equal number of cells (10^6 cells) was inoculated onto liquid Christensen's media in a 10-ml medical tube (SPL Life Sciences, Gyeonggi, Korea). The tubes were incubated at 30°C in a shaking incubator for 1–3 days and photographed daily.

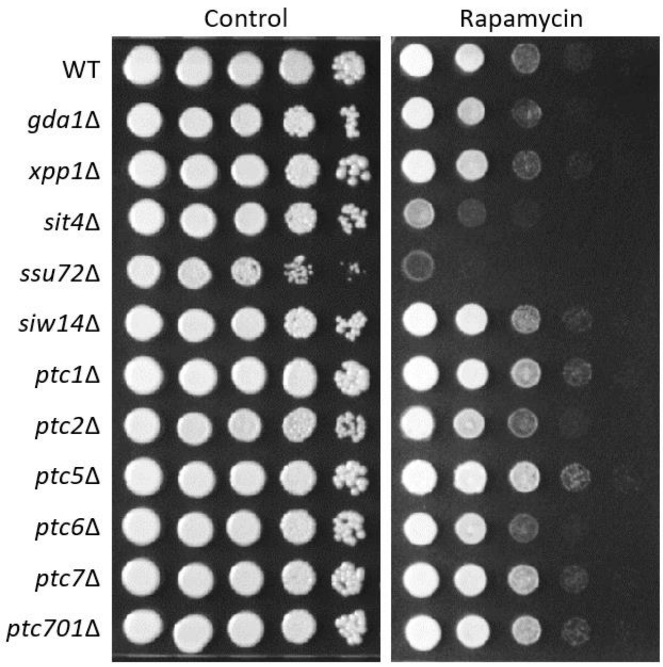

Supplementary Figure 13. Rapamycin susceptibility of *gda1*Δ, *xpp1*Δ, *sit4*Δ, *ssu72*Δ, *siw14*Δ, and PP2C (Type 2C protein phosphatases) mutants. Wild-type (WT) H99S strain and *gda1*Δ, *xpp1*Δ, *sit4*Δ, *ssu72*Δ, *siw14*Δ, and PP2C mutants (*ptc1*Δ, *ptc2*Δ, *ptc5*Δ, *ptc6*Δ, *ptc7*Δ, and *ptc701*Δ) were grown for 16 h at 30°C in liquid YPD medium, serially diluted 10-fold (1 to 10^4), and spotted on YPD agar media containing 10 ng per ml rapamycin. Cells were incubated at 30°C and photographed after 6 days. This is representative of three biologically independent experiments.

References

1 Jung, K. W. *et al.* Systematic functional profiling of transcription factor networks in *Cryptococcus neoformans*. *Nat Commun* **6**, 6757 (2015).

2 Lee, K. T. *et al.* Systematic functional analysis of kinases in the fungal pathogen *Cryptococcus neoformans*. *Nat Commun* **7**, 12766 (2016).

3 Cheon, S. A. *et al.* Unique evolution of the UPR pathway with a novel bZIP transcription factor, Hxl1, for controlling pathogenicity of *Cryptococcus neoformans*. *PLoS Pathog* **7**, e1002177 (2011).

4 Jung, K. W., Kim, S. Y., Okagaki, L. H., Nielsen, K. & Bahn, Y. S. Ste50 adaptor protein governs sexual differentiation of *Cryptococcus neoformans* via the pheromone-response MAPK signaling pathway. *Fungal Genet Biol* **48**, 154-165 (2011).
