# Supplementary material for: Genome-wide functional analysis of phosphatases in the pathogenic fungus Cryptococcus neoformans
Source: Nat Commun. 2020 Aug 24;11:4212. doi: 10.1038/s41467-020-18028-0 (PMC7445287; doi:10.1038/s41467-020-18028-0)
Supplement: Supplementary file 3 — Description of Additional Supplementary Files [file 41467_2020_18028_MOESM3_ESM.pdf]

Description of Additional Supplementary Files

File Name: Supplementary Data 1
Description: List of *Cryptococcus neoformans* phosphatases. A total 139 putative phosphatases genes were listed with each description from FungiDB (https://fungidb.org/fungidb/), genetic locus, InterPro domains (https://www.ebi.ac.uk/interpro/), protein sequence, and SGD (https://yeastgenome.org) description for the *S. cerevisiae* orthologue.

File Name: Supplementary Data 2
Description: BLAST matrix analysis of putative phosphatases in *Cryptococcus neoformans* and other eukaryotes. Comparative BLAST matrix analysis was performed using the Comparative Fungal Genomics Platform (http://cfgp.riceblast.snu.ac.kr).

File Name: Supplementary Data 3
Description: List of putative phosphatases in fungal species. Each sheet shows the list of putative phosphatase genes with InterPro domains and protein sequences in each fungus as follows (Sheet 1, 149 genes in *Candida albicans*; Sheet 2, 151 genes in *Saccharomyces cerevisiae*; Sheet 3, 124 genes in *Schizosaccharomyces pombe*; Sheet 4, 134 genes in *Ustilago maydis*).

File Name: Supplementary Data 4
Description: List of primers used in this study. Primers for constructing *C. neoformans* phosphatase and retromer mutants were listed in Sheet 1. Primers for amplifying selection markers, screening, the quantitative RT-PCR were listed in Sheet 2.

File Name: Supplementary Data 5
Description: *C. neoformans* strains used in this study

File Name: Supplementary Data 6
Description: Putative essential phosphatases, which could not be deleted in this study, in *C. neoformans.* For the list of the putative essential cryptococcal phosphatases, the essentiality of each corresponding orthologue in *S. cerevisiae*, *C. albicans*, and *S. pombe* was indicated with functional description.

File Name: Supplementary Data 7
Description: Complete phenome heat map of *C. neoformans* phosphatase mutants.

File Name: Supplementary Data 8
Description: *In vivo* gene expression of phosphatase genes by NanoString- nCounter® analysis (fold change to WT). The probe information was shown in Sheet 2.

File Name: Supplementary Data 9
Description: Summary of fungal pathogenicity-related phosphatases. The pathogenicity-related phosphatases in *Cryptococcus neoformans* were compared to their orthologues in *Candida albicans*, *Aspergillus fumigatus*, *Fusarium graminearum*, and *Magnaporthe oryzae*.

File Name: Supplementary Data 10
Description: Putative phosphatase regulators in *C. neoformans* and *S. cerevisiae*. The functional annotation of *Saccharomyces* Genome Database (SGD, http://yeastgenome.org) was used to identify putative phosphatase regulatory subunits by BLASTp analysis in *C. neoformans*.
