# Supplementary material for: Genome-wide functional analysis of phosphatases in the pathogenic fungus Cryptococcus neoformans
Source: Nat Commun. 2020 Aug 24;11:4212. doi: 10.1038/s41467-020-18028-0 (PMC7445287; doi:10.1038/s41467-020-18028-0)
Supplement: Supplementary file 14 — Reporting Summary [file 41467_2020_18028_MOESM14_ESM.pdf]

nature research

Corresponding author(s): Yong-Sun Bahn

Last updated by author(s): Jul 25, 2020

Reporting Summary

Nature Research wishes to improve the reproducibility of the work that we publish. This form provides structure for consistency and transparency in reporting. For further information on Nature Research policies, see Authors & Referees and the Editorial Policy Checklist .

Statistics

For all statistical analyses, confirm that the following items are present in the figure legend, table legend, main text, or Methods section.

n/a	Confirmed	
☐	☒	The exact sample size (*n*) for each experimental group/condition, given as a discrete number and unit of measurement
☐	☒	A statement on whether measurements were taken from distinct samples or whether the same sample was measured repeatedly
☐	☒	The statistical test(s) used AND whether they are one- or two-sided<br>*Only common tests should be described solely by name; describe more complex techniques in the Methods section.*
☐	☒	A description of all covariates tested
☐	☒	A description of any assumptions or corrections, such as tests of normality and adjustment for multiple comparisons
☐	☒	A full description of the statistical parameters including central tendency (e.g. means) or other basic estimates (e.g. regression coefficient) AND variation (e.g. standard deviation) or associated estimates of uncertainty (e.g. confidence intervals)
☐	☒	For null hypothesis testing, the test statistic (e.g. *F*, *t*, *r*) with confidence intervals, effect sizes, degrees of freedom and *P* value noted<br>*Give P values as exact values whenever suitable.*
☒	☐	For Bayesian analysis, information on the choice of priors and Markov chain Monte Carlo settings
☒	☐	For hierarchical and complex designs, identification of the appropriate level for tests and full reporting of outcomes
☒	☐	Estimates of effect sizes (e.g. Cohen's *d*, Pearson's *r*), indicating how they were calculated

Our web collection on statistics for biologists contains articles on many of the points above.

Software and code

Policy information about availability of computer code

Data collection	None.
Data analysis	Statistical analyses were performed with GraphPad Prism version 8. NanoString scanning was performed by digital analyser through high resolution (600 fields) option and normalised by nSolver software version 2.5 (provided by NanoString). HPLC analysis data were analysed by using chromatography software (Empower 2, Waters).

For manuscripts utilizing custom algorithms or software that are central to the research but not yet described in published literature, software must be made available to editors/reviewers. We strongly encourage code deposition in a community repository (e.g. GitHub). See the Nature Research guidelines for submitting code & software for further information.

Data

Policy information about availability of data

All manuscripts must include a data availability statement. This statement should provide the following information, where applicable:

- Accession codes, unique identifiers, or web links for publicly available datasets
- A list of figures that have associated raw data
- A description of any restrictions on data availability

Data availability. The phosphatase domain and sequence data in Fig 1 were retrieved from FungiDB (https://fungidb.org/fungidb/) and InterPro (https://www.ebi.ac.uk/interpro/). All data to classify the phosphatases in Fig 1a and 1b are available in Supplementary Data 1. Detailed information about phosphatases in S. cerevisiae, S. pombe, C. albicans and U. maydis is listed in Supplementary Data 3. Information about gene IDs and names, strain numbers, and genotypes with signature tag numbers for phosphatase mutant strains used in this study is available in Supplementary Data 5. We provide the whole phosphatase mutant collection via Fungal Genetics Stock Center (FGSC, http://www.fgsc.net/) in U.S.A., Korean Culture Center of Microorganisms (KCCM, http://www.kccm.or.kr/) and Korean Collection for Type Cultures (KCTC, https://kctc.kribb.re.kr/) in South Korea. The whole phenome data for phosphatase mutants are available in the Cryptococcus neoformans Phosphatase Phenome Database (http://phosphatase.cryptococcus.org). Integrated phenome data of transcription factor, kinase, and phosphatase mutants in C. neoformans in this and previous studies4,5 are available in the Cryptococcus neoformans Phenome Gateway Database (http://

www.cryptococcus.org/), in which individual gene/protein information is linked to that of FungiDB. The whole NanoString-nCounter® analysis data for in vivo phosphatase gene expression and probe information are available in Supplementary Data 8. The source data underlying Supplementary Fig. 7b, 11b, 11d, 11f and 11h are provided as a Source data file.

Field-specific reporting

Please select the one below that is the best fit for your research. If you are not sure, read the appropriate sections before making your selection.

- [x] Life sciences
- [ ] Behavioural & social sciences
- [ ] Ecological, evolutionary & environmental sciences

For a reference copy of the document with all sections, see nature.com/documents/nr-reporting-summary-flat.pdf

Life sciences study design

All studies must disclose on these points even when the disclosure is negative.

Sample size	Three mice were used in each group (each independent mutant for a single phosphatase gene; two independent mutants for a phosphatase gene were tested) for STM study. We chose to perform the STM study on three mice because it would result in a statistically significant outcome, whereas two mice would not. Also, we didn't use more than three mice because of the cost and ethical reasons.
Data exclusions	None.
Replication	For quantitative PCR and BBB crossing/adhesion assays, three biologically independent experiments with triplicates were performed. We indicated all data points in figures. All attempts at replication were successful.<br><br>For diagnostic PCR, Southern blot analysis, spot assay, and virulence factor analysis, we performed more than two independent experiments to confirm the reproducibility. If data show similar patterns, we exhibited one representative image of two independent experiments.
Randomization	The group of mice were chosen at random.
Blinding	The investigators who prepared the STM study with fungal cells performed non-blinding experiment during data collection and analysis, because the investigators should know the mutant strains in each STM group and synchronize the cell numbers. But, the investigators who conducted the mouse study performed blinding experiment during fungal infection and mouse organ harvest. All experiments were performed without any discrimination.

Reporting for specific materials, systems and methods

We require information from authors about some types of materials, experimental systems and methods used in many studies. Here, indicate whether each material, system or method listed is relevant to your study. If you are not sure if a list item applies to your research, read the appropriate section before selecting a response.

Materials & experimental systems

n/a	Involved in the study	
x		Antibodies
	x	Eukaryotic cell lines
x		Palaeontology
	x	Animals and other organisms
x		Human research participants
x		Clinical data

Methods

n/a	Involved in the study	
x		ChIP-seq
x		Flow cytometry
x		MRI-based neuroimaging

Eukaryotic cell lines

Policy information about cell lines

Cell line source(s)	Human brain microvascular endothelial cell (HBMEC) line (hCMEC/D3 cell line, Merck)
Authentication	None.
Mycoplasma contamination	Not tested for mycoplasma contamination
Commonly misidentified lines (See ICLAC register)	None.

Animals and other organisms

Policy information about studies involving animals; ARRIVE guidelines recommended for reporting animal research

Laboratory animals	Seven-week-old female A/J mice (Japan SLC, Inc.)<br><br>The mice were maintained with free access to food and water under a 12-h light and 12-h dark cycle, with the light cycle beginning at 7:00 a.m. The temperature and humidity ranges of housing condition are controlled at 22-23°C and 50-60%, respectively.
Wild animals	None.
Field-collected samples	None.
Ethics oversight	Animal care and all experiments were conducted in accordance with the ethical guidelines of the Institutional Animal Care and Use Committee (IACUC) of Yonsei University. The Yonsei University IACUC approved all of the vertebrate studies.

Note that full information on the approval of the study protocol must also be provided in the manuscript.
